# Investigation of bacterial neuraminidase inhibition of xanthones bearing geranyl and prenyl groups from *Cratoxylum cochinchinense*

**DOI:** 10.3389/fchem.2023.1245071

**Published:** 2023-08-09

**Authors:** Jeong Yoon Kim, Zuo Peng Li, Gihwan Lee, Jeong Ho Kim, Abdul Bari Shah, Yong Hyun Lee, Ki Hun Park

**Affiliations:** ^1^ Department of Pharmaceutical Engineering, Institute of Agriculture and Life Science (IALS), Gyeongsang National University, Jinju, Republic of Korea; ^2^ State Key Laboratory Basis of Xinjiang Indigenous Medicinal Plants Resource Utilization, Xinjiang Technical Institute of Physics and Chemistry, Chinese Academy of Sciences, Urumqi, China; ^3^ Division of Applied Life Science (BK21 Four), Institute of Agriculture and Life Science (IALS), Gyeongsang National University, Jinju, Republic of Korea

**Keywords:** bacterial neuraminidase inhibitors, *Cratoxylum cochinchinense*, new xanthones, slow-binding competitive inhibitor, molecular dynamics simulations

## Abstract

**Introduction:** The root of *Cratoxylum cochinchinense* has been widely used as Chinese folk medicine to cure fevers, burns, and abdominal complications because it contains various bioactive metabolites such as xanthones, triterpenes, and flavonoids. In this study, we estimated bacterial neuraminidase inhibition with a series of xanthones from *C. cochinchinense*. BNA has connected to various biological functions such as pathogenic bacteria infection inflammatory process after infection and biofilm formation.

**Methods:** The identification of xanthones **(1–6)** bearing geranyl and prenyl groups was established by spectroscopic data using UV, IR, NMR, and HREIMS. BNA inhibitory modes of isolated xanthones were investigated by Double-reciprocal plots. Moreover, the competitive inhibitor was evaluated the additional kinetic modes determined by kinetic parameters (*k*_3_, *k*_4_, and *K*_i_^app^). The molecular docking (MD) and molecular dynamics simulations (MDS) studies also provided the critical information regarding the role of the geranyl and prenyl groups against BNA inhibition.

**Results:** A series of xanthones **(1–6)** appended prenyl and geranyl groups on the A-ring were isolated, and compounds **1–3** were shown to be new xanthones. The analogues within this series were highly inhibited with excellent affinity against bacterial neuraminidase (BNA). A subtle change in the prenyl or geranyl motif affected the inhibitory potency and behavior significantly. For example, the inhibitory potency and binding affinity resulting from the geranyl group on C4: xanthone **1** (IC_50_ = 0.38 μM, *K_A_* = 2.4434 × 10^5^ L·mol^−1^) were 100-fold different from those of xanthone **3** (IC_50_ = 35.8 μM, *K_A_* = 0.0002 × 105 L·mol^−1^). The most potent compound **1** was identified as a competitive inhibitor which interacted with BNA under reversible slow-binding inhibition: *K*_i_^app^ = 0.1440 μM, *k*_3_ = 0.1410 μM^−1^s^−1^, and *k*_4_ = 0.0203 min^−1^. The inhibitory potencies (IC_50_) were doubly confirmed by the binding affinities (*K_A_*).

**Discussion:** This study suggests the potential of xanthones derived from *C. cochinchinense* as promising candidates for developing novel BNA inhibitors. Further research and exploration of these xanthones may contribute to the development of effective treatments for bacterial infections and inflammatory processes associated with BNA activity.

## 1 Introduction

N-Acetylneuraminic acid (Neu5Ac, sialic acid) is a nine-carbon sugar and is present as terminal residue on glycans, glycoproteins, and glycolipids (Lewis and Lewis, 2012; Kooner et al., 2019). This linkage of Neu5Ac plays a crucial role in cellular recognition in both pathological and physiological processes (Zhang et al., 2019). Bacterial neuraminidase (BNA) is an exoenzyme to the hydrolase terminal sialic acid unit with an α(2→3) or α(2→6) linkage (Vimr et al., 2004). BNA is involved in many biological processes such as pathogenic bacteria infection, inflammation after infection, and biofilm formation (Soong et al., 2006; Nita-Lazar et al., 2015). Some pathogenic bacteria such as *Pseudomonas aeruginosa* target the cell with removal of the terminal sialic acid unit of the glycan (Khatua et al., 2014; Sanchez et al., 2021). The sialic acid linkage is also essential to open the inflammatory cascade after bacterial infection through the generation of cytokines, which leads to sepsis (Casey, 2000). Thus, bacterial infection and subsequent inflammatory processes could be controlled by the inhibition of the BNA enzyme (Kuroiwa et al., 2009; Fernández-Arjona et al., 2019). In particular, BNA is one of the key factors responsible for biofilm formation together with quorum sensing, which comprises encapsulation of the population of bacteria within the extra-cellular matrix (Domenech et al., 2012). The biofilm protects the microorganisms not only from alterations in the pH, osmolarity, and nutrient scarcity but also prevents antibiotics from accessing the bacterial communities (Fux et al., 2005).

*Cratoxylum cochinchinense* belongs to the family of Hypericaceae and grows naturally in Southern Asian countries (Mustaqim and Amboupe, 2020). The roots and bark are both used in traditional Chinese medicine for the treatment of fevers, burns, and abdominal complications (Thu et al., 2017; Tan et al., 2022). Xanthones, caged xanthones, triterpenoids, and flavonoids have been reported as bioactive constituents of *C. cochinchinense* (Nguyen and Harrison, 1999; Mahabusarakam et al., 2006; Xiong et al., 2014; Li et al., 2018a). The most abundant metabolites in the plant, the xanthones, have diverse biological functions such as antioxidant (Panda et al., 2013), antimalarial (Riscoe et al., 2005), antibacterial (Siridechakorn et al., 2012), and anti-HIV effects (Groweiss et al., 2000). In particular, the xanthones extracted from the roots had significant inhibitory potential against protein tyrosine phosphatase 1B (PTP1B) and α-glucosidase, which are associated with diabetes and obesity (Li et al., 2018b).

In the course of disclosing the metabolites of the target plant, we isolated a series of xanthones with prenyl and geranyl groups appended to the A-ring. In this study, we identified the structures of these compounds, which included three new compounds (**1**–**3**), by spectroscopic analysis. Their biological function regarding the inhibition of bacterial neuraminidase was experimentally investigated to determine their kinetics and binding affinity. In addition, *in silico* experiments, including molecular dynamic simulations, were conducted and enabled us to fully disclose their inhibitory properties.

## 2 Materials and methods

### 2.1 Plant materials

*Cratoxylum cochinchinese* (Lour.) Blume was collected from the Guangdong province of China. The collection of voucher specimens (No. 22719, voucher specimen) was deposited by Dr. Huangu Ye at the Herbarium of the South China Botanical Garden (Chinese Academy of Science, Guangdong province, China). The root barks of *C. cochinchinense* were dried and prepared to isolate the metabolites.

### 2.2 Chemicals and instruments

Organic solvents such as methanol, acetone, ethyl acetate, chloroform, and hexane, which were used for extraction and purification, were purchased from Duksan (Gyeonggi-do, South Korea). The metabolites isolated from the root bark of *C. cochinchinense* were chromatographed on an Acclaim Polar Advantage II column (250 × 20 mm, 5 μm, Thermo Scientific™, MA, United States) and ODS-AQ (250 × 30 mm, 5 μm, YMC Co. Ltd., Tokyo, Japan) using prep-LC (Forte-R, YMC Co. Ltd.). Normal phase (NP F254) and reversed phase (RP-18 F254) TLC plates were purchased from Merck (Kenilworth, NJ, United States). Sephadex LH-20 was acquired from GE Healthcare (Chicago, IL, United States) for purification of the isolated compounds. The chemical structures of the isolated metabolites were identified by ^1^H, ^13^C-NMR, COSY, HMBC, and HMQC spectroscopy using an NMR spectrometer (AM 500 MHz, Bruker Bioscience Co., Billerica, MA, United States). Electron ionization and high-resolution mass spectra (JEOL Ltd., Tokyo, Japan) were recorded to confirm the chemical mass and formula. Infra-red (IR) spectrum was obtained from Spectrum two (Perkin Elmer, Waltham, MA, United States). Melting points are uncorrected. Optical rotation was measured on an Automatic polarimeter P-2000 (JASCO, Tokyo, Japan). Ultra-violet (UV) spectrum and fluorescence were measured using an iD3 spectrophotometer (Molecular Devices, San Jose, CA, United States). Neuraminidase from *Clostridium perfringens*, 2'-(4-methylumbelliferyl)-α-_D_-*N*-acetyl-neuraminic acid sodium salt hydrate, and dimethyl sulfoxide were purchased from Sigma Aldrich (St. Louis, MA, United States). Gentisein, used as a positive control in the enzyme inhibition, was purchased from Chemfaces (Wuhan, Hubei, PRC). Water, methanol, and acetonitrile used for MPLC was analytical grade (Honeywell Inc., Charlotte, NC, United States).

### 2.3 Isolation of xanthones from the root bark of *Cratoxylum cochinchinense*

The dried root bark of *C. cochinchinense* (0.5 kg) was extracted with methanol (20 L) for 2 weeks at room temperature. The methanol extract was evaporated to obtain the crude residues (68 g). The residues were dissolved in water and separated using organic solvents with different polarities to give *n*-hexane, chloroform, ethyl acetate, and water layers. In solvent fractionation with different polarities, the chloroform was found to be the appropriate solvent regarding alkylated xanthones. Thus, the chloroform layer (18 g) was repeatedly subjected to LC/Forte-R MPLC over an ODS-AQ column (YMC Co. Ltd., 250 × 30 mm, 5 µm) and eluted with a methanol/water gradient system containing from 70% to 100% methanol to collect eight fractions (Fr. 1–8) at a flow rate of 20 mL/min. Fr. 2 (1.4 g) was fractionated by monitoring the separated UV peaks at 254 nm over an Acclaim Polar Advantage II column (Thermo Scientific, 250 × 20 mm, 5 μm) by increasing the portion of methanol in water (v/v, 80% → 100%) to afford five subfractions (Fr. 2A ∼ Fr. 2E). One of these subfractions (Fr. 2C, 50 mg) was purified on Sephadex LH-20 with 95% methanol to obtain compound **1** (23 mg). Fr. 5 (980 mg) was chromatographed to yield a mixture of compounds **2** and **3**, which was subsequently separated via the recycle mode of MPLC eluting with 85% acetonitrile in isocratic mode to yield compounds **2** (19 mg) and **3** (10 mg), respectively. Injection of Fr.6 (2.3 g) into the Acclaim Polar Advantage II column followed by elution with an increasing ratio of acetonitrile in water (*v*/*v*) from 70% to 100% in a gradient manner, not only yielded a mixture of compounds **4** and **5** with 80% acetonitrile, but also compound **6** (21 mg), which was obtained by the continuous chromatography of Fr. 6 with 95% acetonitrile. The mixture enriched with compounds **4** and **5** was separated on Sephadex LH-20 using 90% methanol to yield compounds **4** (12 mg) and **5** (8 mg).

### 2.4 Bacterial neuraminidase inhibitory activity

Fluorometric assays were used to determine the bacterial neuraminidase inhibitory activity of the isolated xanthones (**1**–**6**) and gentisein as the basic skeleton of xanthone. The xanthones used in the experiments were dissolved in DMSO to prepare 8 mM (stock) and then diluted by half. 2’-(4-methylumbelliferyl)-α-_D_-*N*-acetylneuraminic acid sodium salt hydrate, used as a substrate, was hydrolyzed by neuraminidase from *C. perfringens* (BNA, EC 3.2.1.18) to form N-acetylneuraminic acid and 4-methylumbelliferone. These two compounds undergo blue fluorescence (FS) emission at 365 nm upon excitation at 450 nm. The reactant for evaluation of BNA inhibitory activity was tested in sodium acetate buffer (pH 5.0) with and without 10 µL of candidate enzyme inhibitors, 20 µL of the fluorogenic substrate (100 μM, *K*_m_), and 10 µL of BNA (0.02 unit/mL) on a black flat-bottom 96-well plate. The FS intensities (RFU/min) of the reaction mixture were recorded using an iD3 spectrophotometer every 1 min for 20 min. The enzyme inhibitory potencies of the xanthones was investigated by calculating the half-maximal inhibitory concentrations against BNA derived from the following equation: [(FS intensities of control)—(FS intensities of xanthone treatment)]/(FS intensities of control).

### 2.5 Determination of enzyme kinetic modes

The enzyme kinetics of the isolated xanthones (**1**–**6**) was studied to investigate the reversibility and inhibitory mechanism against BNA. The enzyme reversibility was confirmed by treating different concentrations of BNA (0.01, 0.02, and 0.04 unit/mL) with the inhibitors (0.5 × IC_50_, IC_50_, and 2 × IC_50_) while maintaining the other conditions such that they were the same as in the BNA inhibition experiments. The Lineweaver-Burk plot was calculated from the double reciprocal of different concentrations of substrate (0.5 × *K*_m_, *K*_m_, and 2 × *K*_m_) and FS intensities (RFU/min) to evaluate the inhibition mechanism of the isolated xanthones as BNA inhibitors. Dixon plots were derived from different concentrations of inhibitors (0.5 × IC_50_, IC_50_, and 2 × IC_50_) and the reciprocal of the FS intensity (RFU/min) to examine the inhibition constants (*K*_i_) against the BNA inhibitors. Furthermore, xanthone **1** as a competitive inhibitor (0.4 μM, IC_50_) and BNA (0.02 unit/mL) were preincubated for different periods of time (0, 5, 15, 30, 45, 60, and 75 min) in the dark at 37°C. Then, the time-depending BNA inhibitory activity was confirmed by measuring the FS intensities of the mixture immediately after treatment with the substrate (100 μM, *K*_m_). In similar approaches, different concentrations of the competitive inhibitor (0, 0.2, 0.4, and 0.8 μM) were preincubated with a fixed concentration of BNA (0.02 unit/mL) in buffer for 10 min in intervals of 2 min. After preincubation, the substrate was added to obtain the kinetic parameters (*k*_3_, *k*_4_, *K*_i_^app^, and *K*_obs_) derived from the following equations (1–3).

$$v/v_{0}=\exp\left(-K_{\text{o}\text{b}\text{s}}t\right)$$ (1)

$$K_{obs}=k_{4}\left(1+\left[I\right]/K_{i}^{app}\right)$$ (2)

$$K_{i}^{app}=k_{4}/k_{3}$$ (3)

where *v* and *v*_0_ are the FS intensities with or without preincubation (0 min); *K*_obs_ is the apparent pseudo-first-order rate constant; *K*_i_^app^ is the apparent inhibition constant; *k*_3_ and *k*_4_ are the rate of the forward and reverse reactions to form the enzyme-inhibitor complex (Cha, 1975; Morrison, 1982).

### 2.6 Measurements of binding affinity between BNA and their inhibitors

The binding affinity between BNA and the inhibitors (xanthones) was determined using the FS quenching method (Ryu et al., 2014). In the absence of a substrate, the different concentrations of inhibitors (0, 0.2, 0.4, 0.8, 1.6, and 3.2 µM) and the BNA (0.02 unit/mL) on the 96-well plate were measured using the wavelength scan mode of the spectrophotometer. The excitation wavelength was fixed at 275 nm, and the emission wavelength was 300–400 nm in intervals of 2 nm. The binding affinity parameters such as *K*_SV_, *K*_A_, and *n* were derived using a Sigma plot of the scanned FS curve for different concentrations of the isolated xanthones with reference to the equation in Section 3.4.

### 2.7 Molecular docking experiments

The crystal structure of *Clostridium perfringens* neuraminidase (PDB ID: 5TSP) with resolution of 1.24 Å was obtained from the RCSB Protein Data Bank (www.rcsb.org), complexed with the inhibitor CHES (Lee et al., 2017). Subsequently, the protein was prepared by enabling the *Clean Protein* tool in the Discovery Studio (DS) v2019. The water molecules and heteroatoms were removed and the protein was minimized employing the *Minimize and Refine Protein* module available with DS. For the molecular docking (MD) study, the three-dimensional (3D) structures of three inhibitors, compounds **1**, **2**, **4**, and **6**, were prepared using the *sketching tool* (PubChem Sketcher v2.4) after which their geometry was optimized by employing the *Avogadro* program. The binding modes of the three inhibitors at the *Clostridium perfringens* neuraminidase docking site were explored by employing GOLD Suite 5.2.2 (Cambridge Crystallographic Data Center, United Kingdom), which uses genetic algorithms (Verdonk et al., 2003). Correspondingly, the docking site was defined within 10 Å around the catalytic site and allosteric site with reference to the quercetin docking study as reported previously (Wagle et al., 2018). During the MD computations, each ligand was allowed to generate 10 conformations, while the default values of the other parameters were retained. The best binding modes of the compounds were selected based on the highest GOLD fitness score.

### 2.8 Molecular dynamics simulation experiments

The 5 ns molecular dynamics simulations (MDS) were performed for the selected bonding mode. MDS is a computer simulation approach that enables the motions of the molecules of interest to be observed as a function of time by calculating the motion of the atoms in the molecules according to Newton’s laws of motion (Collier et al., 2020). MDS provides an opportunity to conduct various studies such as molecular design, for example, drug design and protein design, with the advantage of enabling the interaction between a target protein and a new drug candidate to be investigated in detail at the atomic level. Before simulation, the structure was optimized through energy minimization to avoid stereoscopic collisions or the presence of inappropriate geometries. After energy minimization, two-step equilibration was performed. The first step of equilibration was performed in an NVT ensemble (constant number of particles, volume, and temperature) at 300 K for 100 ps using a V-rescale thermostat. The second phase of equilibration was performed in the NPT ensemble (constant particle count, pressure, and temperature) for 100 ps. Pressure of 1 bar was applied to the system using a Parrinello-Rahman barostat. During the equilibration process, the protein backbone was suppressed while the solvent molecules moved along with the counterions. MDS was performed at 300 K and 1 bar for 5 ns with the NPT equilibrium structure as the starting structure.

### 2.9 Statistical analysis

The measurements of BNA inhibitory activities, enzyme kinetics, and binding affinities were made in triplicate. The mean, deviations, and *p*-values (<0.05) of the results were expressed using SigmaPlot ver. 10.0 (Systate Software Inc., Chicago, IL, United States).

## 3 Results and discussion

### 3.1 Isolation and identification of xanthones

**FIGURE 1:**
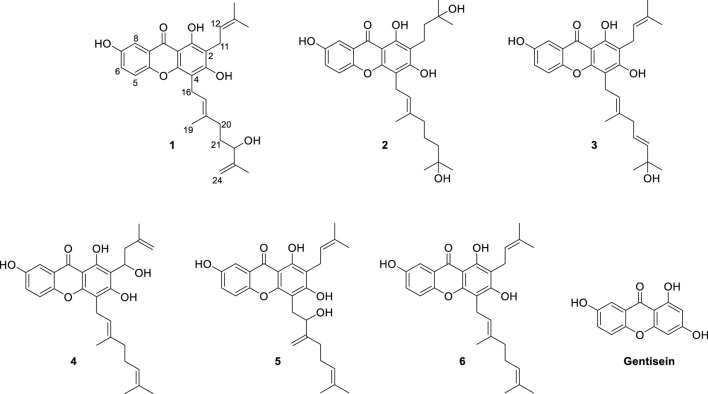
Chemical structures of the isolated xanthones (**1**–**6**) from the root barks of *Cratoxylum cochinchinense* and their basic skeleton (gentisein).

Our aim was to isolate a series of xanthones intended for bacterial neuraminidase inhibition from the methanol extract of the roots of *C. cochinchinense*. The main constituents of the extract are series of xanthones (**1**–**6**) bearing geranyl and prenyl groups on the A-ring as shown in Figure 1. These xanthones were purified over Sephadex LH-20 and octadecyl-functionalized silica gel, as delineated in the experimental section. The isolated xanthones (**1**–**6**) were identified as the known species of cratoxanthone A (**4**), cratoxanthon F (**5**), and cochinechinone A (**6**) together with three new xanthones named as cratoxanthones G-I (**1**–**3**). The detailed spectroscopic data were arranged in Table 1.

**TABLE 1:** ^1^H-NMR and ^13^C-NMR data of new compounds **1**–**3** (500 MHz).

No	1 (Acetone-*d*_6_)		2 (Acetone-*d*_6_)		3 (CDCl_3_)	
	*δ*_H_, multi, (*J*, Hz)	*δ*_C_	*δ*_H_, multi, (*J*, Hz)	*δ*_C_	*δ*_H_, multi, (*J*, Hz)	*δ*_C_
1		158.2		158.2		158.1
2		108.6		111.5		108.7
3		160.9		161.2		160.9
4		105.2		106.6		105.1
4a		153.0		153.1		153.1
5	7.21, d (9.0)	118.8	7.48, d (9.0)	118.8	7.23, d (9.0)	118.6
5a		152.4		150.0		150.2
6	7.12, dd (9.0, 3.0)	124.0	7.35, dd, (9.0, 3.0)	124.0	7.16, dd (9.0, 3.0)	124.1
7		150.2		153.7		152.5
8	7.42, d (3.0)	108.8	7.59, d (3.0)	108.4	7.49, d (3.0)	108.8
8a		120.5		120.7		120.4
9		180.8		180.7		180.8
9a		103.1		102.5		103.1
11	3.35, d (7.0)	21.6	2.84, br t (6.9)	16.5	3.39, d (7.0)	21.6
12	5.20, t (7.0)	121.5	1.79, br t (6.9)	41.6	5.25, br t (7.0)	121.5
13		135.6		70.6		135.4
14	1.77, s	17.9	1.29, s	28.7	1.82, s	17.7
15	1.69, s	25.8	1.29, s	28.7	1.75, s	25.9
16	3.45, d (7.0)	21.6	3.57, d (7.1)	21.6	3.49, d (7.0)	21.8
17	5.30, t (7.0)	122.6	5.28, t (7.1)	122.6	5.29, br t (7.0)	122.7
18		136.7		134.7		135.8
19	1.82, s	16.2	1.89, s	15.3	1.85, s	16.3
20	2.05, m	35.9	1.96, m	40.0	2.71, d (7.0)	42.2
21	1.59, m	32.7	1.49, m	22.4	5.63, m	125.1
22	4.00, m	75.9	1.33, m	43.2	5.62, m	139.4
23		147.1		69.3		71.2
24	4.72, 4.87, s	111.2	1.09, s	28.9	1.30, s	29.7
25	1.62, s	17.7	1.09, s	28.9	1.30, s	29.7
1-OH	12.97, s		13.28, s		13.00, s	

**FIGURE 2:**
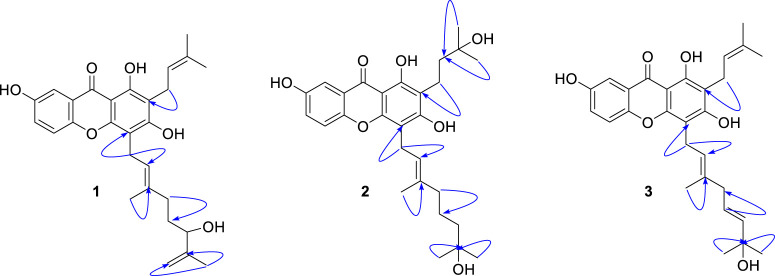
HMBC correlation (H→C) of new compounds **1–3**.

Compound **1** was obtained as a yellowish solid with the molecular formula C_28_H_32_O_6_ and 13 degrees of unsaturation, as established by HREIMS, with *m*/*z* 464.2197 [M]^+^ (calcd. 464.2199). The IR (3,425, 2,920, and 1,660 cm^−1^, KBr) and UV (λ_max_ 230, 280, and 360 nm) were agree to typical alkylated xanthone. DEPT experiments indicated that the 28 carbon atoms consisted of 4 methylene (sp^3^), 1 methylene (sp^2^), 1 methine (sp^3^), 5 methine (sp^2^), 4 methyl, and 13 quaternary carbon atoms. Among the 13 degrees of unsaturation, the ten double bonds were identified. Thus, the three degrees of unsaturation were ascribed to three rings in xanthone. The proton within the hydroxyl group on C-1 (*δ*_C_ = 158.2) was observed at *δ*_H_ = 12.97. This is consistent with the formation of a hydrogen bond between the C-1(OH) and the carbonyl group (*δ*_C_ = 180.8). The presence of the 1,1-dimethylallyl (prenyl) group was deduced from the successive connectivities between H-11 (*δ*_H_ = 3.35, d, *J* = 7.0 Hz) to H-14/15 (*δ*_H_ = 1.77/*δ*_H_ = 1.69, 6H) on the COSY spectrum. The HMBC correlation of H-11 (*δ*_H_ = 3.35) with C-2 (*δ*_C_ = 108.6) proved the location of prenyl group. The presence of the 3-methyl-6-hydroxy-7-methylene-2-octenyl (geranyl) group was deduced from successive connectivities between H-16 (*δ*_H_ = 3.45, d, *J* = 7.0 Hz) to H-17 (*δ*_H_ = 5.3, t, *J* = 7.0 Hz), H-19 (*δ*_H_ = 1.82, s), H-20 (*δ*_H_ = 2.05, m), H-21 (*δ*_H_ = 1.59, m), H-22 (*δ*_H_ = 4.00, m), and H-24a/b (*δ*_H_ = 4.72, 4.87, s). The position of hydroxyl moiety in geranyl group was proved HMBC correlation between oxygenated carbon C-22 (*δ*_C_ = 75.9) and exomethylene H-24a/b. The strong HMBC correlation of H-16 with C-4 (*δ*_C_ = 105.2) proved the position of geranyl group on C-4 (Figure 2). The presence of the B-ring was confirmed by the ABX pattern among H-5 (*δ*_H_ = 7.21, d, *J* = 9.0 Hz), H-6 (*δ*_H_ = 7.12, dd, *J* = 9.0 and 3.0 Hz), and H-8 (*δ*_H_ = 7.42, d, *J* = 3.0 Hz). The HMBC correlations in Figure 2 matched with the suggested structure of **1** properly. The specific rotation value ([α]) was measured to be +11.3 (0.1, MeOH), but its absolute configuration was not confirmed. Thus, compound **1** was identified as (*E*)-1,3,7-trihydroxy-4-(6-hydroxy-3,7-dimethylocta-2,7-dien-1-yl)-2-(3-methylbut-2-en-1-yl)-9*H*-xanthen-9-one, and referred to as cratoxanthone G.

The HREIMS of compound **2** revealed a molecular ion *m*/*z* 484.2461 [M]^+^ (calcd. 484.2461) consistent with a molecular formula of C_28_H_36_O_7_. The IR and UV spectra were similar to those of **1**. The B-ring was deduced by interpretation of NMR spectra which has very similar with pattern of **1**. The 1-hydroxy-1-methyl butanyl group was confirmed by COSY correlation of H-11 (*δ*_H_ = 2.84, t) to H-12 (*δ*_H_ = 1.79, t), and HMBC correlation of H-12 with the oxygenated carbon C-13 (*δ*_C_ = 70.6). HMBC correlation of H-11 with C-2 (*δ*_C_ = 111.5) proved the location of this prenyl group. The presence of the 7-hydroxy-3,7-dimethyl-2-octenyl (geranyl) group was deduced from the successive connectivities between H-16 (*δ*_H_ = 3.57, d) and H-22 (*δ*_H_ = 1.33, m) and the HMBC correlation of oxygenated carbon C-23 (*δ*_C_ = 69.3) with H-22. HMBC correlation of H-16 (*δ*_H_ = 3.57, d) with C-4 (*δ*_C_ = 106.6) proved the location of geranyl group. Thus, compound **2** was identified as (*E*)-1,3,7-trihydroxy-4-(7-hydroxy-3,7-dimethyloct-2-en-1-yl)-2-(3-methylbut-2-en-1-yl)-9*H*-xanthen-9-one, and referred to as cratoxanthone H.

The molecular formula of compound **3** was identified as C_28_H_32_O_6_ by HREIMS with *m*/*z* 464.2197 [M]^+^ (calcd. 464.2199). The IR and UV spectra were similar to those of **1**. The B-ring and prenyl group on C-ring was elucidated by interpretation of NMR spectra which has similar pattern with compound **1**. The 7-hydroxy-3,7-dimethyl-2,5-octadienyl (geranyl) group was confirmed by successive connectivities from H-16 (*δ*_H_ = 3.49, d, *J* = 7.0 Hz) to H-22 (*δ*_H_ = 5.62, m) in COSY spectrum and HMBC correlation of H-22 with oxygenated carbon C-23 (*δ*_C_ = 71.2). The location of geranyl group was proved by HMBC correlation of H-16 with C-4 (*δ*_C_ = 105.1). Thus, compound **3** was identified as 1,3,7-trihydroxy-4-[(2*E*,5*E*)-7-hydroxy-3,7-dimethylocta-2,5-dien-1-yl]-2-(3-methylbut-2-en-1-yl)-9*H*-xanthen-9-one, and referred to as cratoxanthone I.

### 3.2 Bacterial neuraminidase inhibitory activity of the isolated xanthones

**FIGURE 3:**
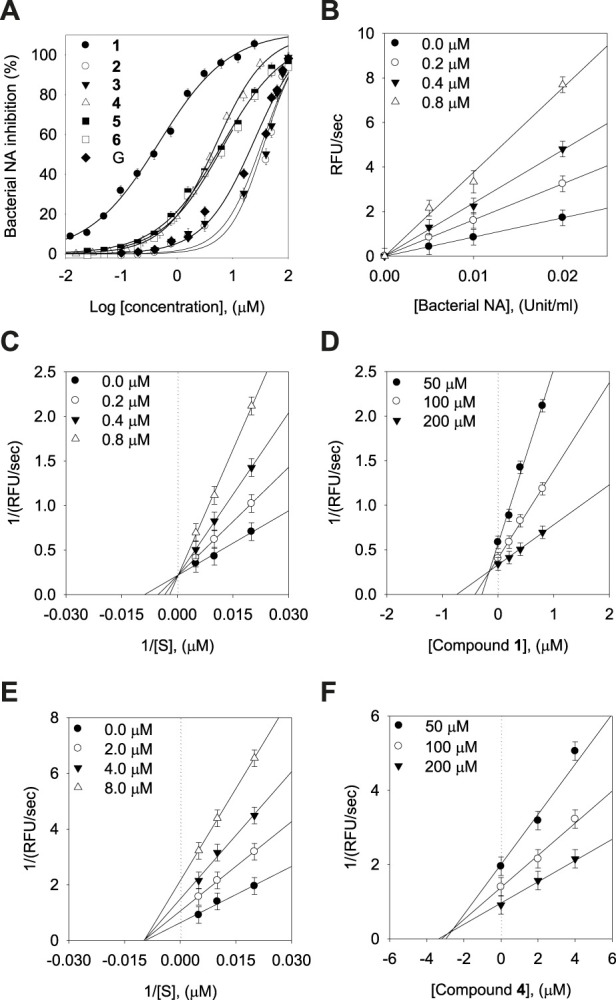
Effects of the isolated xanthones on bacterial neuraminidase (BNA) activities **(A)** Dose-dependent inhibition of BNA inhibition by the isolated compounds (**1**–**6**) and gentisein (basic xanthone skeleton), **(B)** Determination of the reversibility of compound **1** on BNA, **(C)** Lineweaver-Burk plots of compound **1**, **(D)** Dixon plots of compound **1**, **(E)** Lineweaver-Burk plots of compound **4**, **(F)** Dixon plots of compound **4**.

The inhibitory capacities of the isolated xanthones were investigated against bacterial neuraminidase (BNA) from *Clostridium perfringens*. All compounds exhibited a dose-dependent inhibitory effect on the BNA enzyme (IC_50_ = 0.38 ∼ 38.9 µM) as shown in Figure 3A and Table 2. They all have the structural feature comprising an A-ring with prenyl (5 carbons) and geranyl (10 carbons) groups as substituents. The inhibitory capacity was significantly affected by a subtle change in the geranyl and prenyl functionality. Compounds **1**, **3**, and **6** have the same structural feature except for modification of the geranyl motif on C-4. Compound **1**, which bears hydroxyl and exomethylene functionalities on the geranyl motif exhibited 30-fold higher inhibition than compound **6** (IC_50_ = 9.8 µM), which possesses a typical geranyl group. Compound **1** was 100-fold more effective than compound **3** (IC_50_ = 35.8 µM). The role of the geranyl motif in compound **1** with respect to BNA inhibition was additionally explored by conducting a detailed kinetic study to determine the fluorescence (FS) quenching effect and by performing molecular docking experiments (*vide infra*). A comparison of compounds **4** and **6**, which have the same structural feature except for the modification of the prenyl group on C-2, revealed that the inhibitory effect of compound **4** (IC_50_ = 3.8 µM) was 3-fold higher than that of **6**. Compound **6**, which bears the typical prenyl and geranyl groups on C-2 and C-4, was 2-fold more effective relative to the mother compound, gentisein (IC_50_ = 21.4 µM). More detailed bioassays of the isolated xanthones were subsequently conducted. The reversibility of xanthone **1**, the most potent inhibitor, to the BNA enzyme, is shown in Figure 3B, representatively, in which the residual enzyme activity is plotted versus the enzyme concentration for different concentrations of **1**. A family of straight lines passing through the origin at the x-axis is indicative of a reversible inhibitor. All compounds manifested a similar relationship to the enzyme regarding reversibility. The kinetic behaviors of the hydrolysis of sialic acid (a sugar with nine carbon atoms) catalyzed by the BNA enzyme at different concentrations of the compounds were estimated using Lineweaver-Burk and Dixon plots (Figures 3C–F). The compounds (**2**–**6**) produced a family of straight lines with the same intercept on the x-axis (Figures 3E, F, and Supplementary Material). This indicated that these compounds are typical non-competitive inhibitors because *K*_m_ did not change, but *V*_max_ increased. Compound **1** was identified as a competitive inhibitor because a family of straight lines had the same intercept with the y-axis indicating that *V*_max_ did not change (Figures 3C, D), whereas *K*_m_ increased.

**TABLE 2:** Inhibitory effects of the isolated xanthones (**1**–**6**) **on bacterial neuraminidase activities.**

Compounds	Bacterial neuraminidase	
	IC_50_ ^a^, (μM)	Kinetic mode (*K*_i_ ^b^, μM)
**1**	0.38 ± 0.03	Competitive (0.17 ± 0.05)
**2**	38.9 ± 0.6	Non-competitive (40.7 ± 2.8)
**3**	35.8 ± 0.7	Non-competitive (38.2 ± 2.3)
**4**	3.8 ± 0.1	Non-competitive (4.2 ± 0.8)
**5**	9.2 ± 0.2	Non-competitive (9.1 ± 0.5)
**6**	9.8 ± 0.3	Non-competitive (9.6 ± 0.3)
Gentisein^c^	21.4 ± 0.5	NT^d^

All the tested compounds were examined in triplicates.

^a^ IC_50_ values of xanthones represent the 50% enzyme inhibition ratio at the concentrations.

^b^ *K*_i_ means the inhibition constants.

^c^ Gentisein was used as a positive control with the basic xanthone skeleton.

^d^ NT is not tested.

**FIGURE 4:**
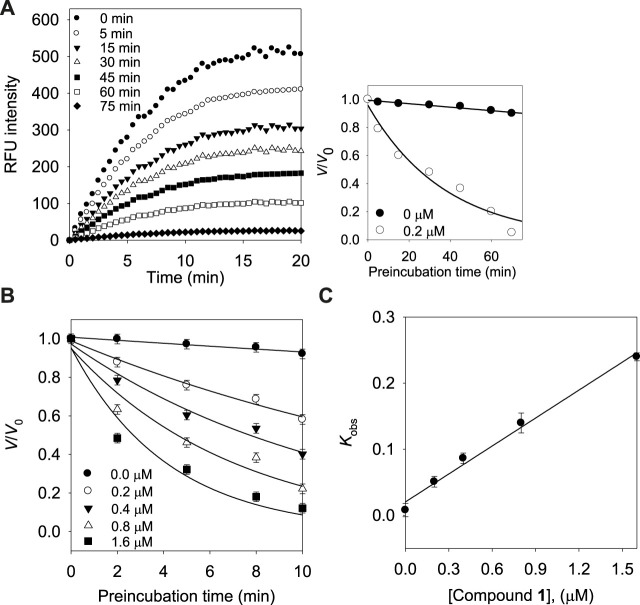
**(A)** Time course of substrate hydrolyzed by BNA in the presence of compound **1** at 0.2 µM. (Inset) Decrease in slopes of the lines of panel **(A)** as a function of time. **(B)** Pre-incubation time dependence of the velocity of an enzyme-catalyzed reaction in the presence of varying concentrations of compound **1**. **(C)** Dependence of the values for *K*_obs_ on the concentrations (0, 0.2, 0.4, and 0.8 µM) of compound **1**.

Subsequently, we investigated the time dependent inhibition of the most potent and competitive inhibitor **1**. This phenomenon was examined by preincubation of the BNA enzyme with the inhibitor over various time points. When the enzyme was preincubated with inhibitor **1** for a period of between 0 and 75 min, a successive decrease in activity was observed (Figure 4A). The BNA enzyme was found to lose no more than its activity up to 75 min of preincubation (Figure 4A insert). At fixed inhibitor concentrations (0, 0.2, 0.4, and 0.8 µM), the residual velocity decayed exponentially with the preincubation times (Figure 4B). These progress curves were fitted to Eq. 1 to determine *v*, *v*_0_, and *K*_obs_. Figure 4C shows a plot that represents the relationship between *K*_obs_ and [I], and indicates a linear dependence of *K*_obs_ on the concentration of inhibitor **1**. The kinetic parameters (*k*_3_, *k*_4_, and *K*_i_^app^) were derived from the plots by fitting the results to Eqs. 2, 3, which gives *K*_i_^app^ = 0.1440 µM, *k*_3_ = 0.1410 µM^−1^s^−1^, and *k*_4_ = 0.0203 min^−1^ (Table 3). Based on the above kinetic observation, we believed inhibitor **1** to be a simple slow-binding inhibitor. Another advantage of these prenyl and geranyl xanthones is that they have much higher lipophilicities (log *p* = 4.42–6.30) than the mother skeleton, gentisein (log *p* = 1.58) which might facilitate penetration of the cell.

**TABLE 3:** Kinetic parameters for slow-binding inhibition of bacterial neuraminidase by compound **1**.

Compounds	*K*_i_^app^ (µM)	*k*_3_ (µM^−1^s^−1^)	*k*_4_ (min^−1^)
**1**	0.1440	0.1410	0.0203

### 3.3 Binding affinity by using fluorescence quenching spectra

**FIGURE 5:**
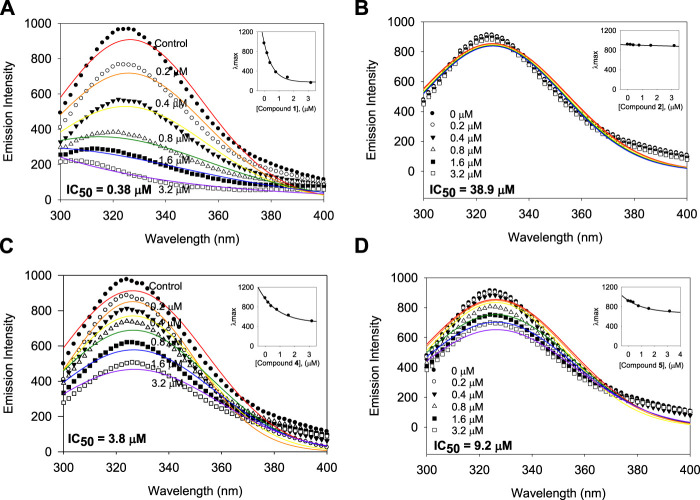
Binding affinity between BNA and the inhibitors **1**, **2**, **4**, and **5** with the fluorescence quenching effects. **(A–D)** Fluorescence emission spectra of the isolated xanthones **1**, **2**, **4**, and **5** at 0, 0.2, 0.4, 0.8, 1.6, and 3.2 μM (Inset) The maximal emission wavelength of xanthones **1**, **2**, **4**, and **5** at the concentrations of 0, 0.2, 0.4, 0.8, 1.6, and 3.2 μM.

A subtle change in the prenyl or geranyl group results in a significant difference in the inhibitory potency of BNA. This made it necessary to corroborate the binding affinity and inhibitory potencies against the enzyme. The BNA enzyme has intrinsic fluorescence originating from the tryptophan (Trp-31, 80, 118, 124, 135, 149, 172, 217, and 264), tyrosine (Tyr-35, 57, 65, 82, 95, 141, 203, 204, and 14 other residues), and phenylalanine (Phe-8, 24, 36, 52, 76, 286, 322, and 352) residues (Supplementary Material). Its intrinsic fluorescence diminishes as a function of the ligand affinity or concentrations (Möller and Denicola, 2002; Ghisaidoobe and Chung, 2014). We investigated the affinity of the geranyl and prenyl xanthones (**1**–**6**) for the BNA enzyme. None of the other components of the assay mixture exhibited significant emission under the measurement conditions. The fluorescence (FS) intensity decreased in proportion to the increasing concentration from 0 to 3.2 µM as shown in Figure 5. However, the degree of decrease is also significantly correlated with the inhibitory potency. For example, the FS of inhibitor **1** (IC_50_ = 0.38 µM) decreased dramatically as its concentration increased, but the FS intensity of inhibitor **2** (IC_50_ = 38.9 µM) did not decrease (Figures 5A, B). The Stern-Volmer constant (*K*_SV_) and affinity constant (*K*_A_) were analyzed by the following equation: log [(*F*_0_-*F*)/*F*] = log*K*_A_ + *n*log [Q]_f_, where *F*_0_ and *F* are the fluorescence intensities in the absence or presence of the inhibitors; *K*_A_ is the affinity constant; *n* is the number of binding sites between the enzyme and quencher (xanthone); [Q]_f_ is the concentration of the quencher (xanthone) (Lakowicz, 1999; Wang et al., 2007). The *K*_A_ values of the inhibitors were ranked in the following order: **1** (IC_50_ = 0.38 µM) > **4** (IC_50_ = 3.8 µM) > **6** (IC_50_ = 9.8 µM) > **2** (IC_50_ = 38.9 µM), which is in agreement with the order of their inhibitive potencies (IC_50_), as shown Figure 5 and Table 4.

**TABLE 4:** Fluorescence quenching effects of the isolated xanthones (**1**–**6**) and gentisein on bacterial neuraminidase.

Compounds	*K*_SV_ ^a^ (× 10^5^ L·mol^−1^)	*R* ^2^	*K*_A_ ^b^ (× 10^6^ L·mol^−1^)	*n^c^*	*R* ^2^
**1**	1.5792	0.9955	0.24434	1.2897	0.9956
**2**	0.0097	0.9985	0.00002	0.1796	0.9997
**3**	0.0100	0.9967	0.00002	0.2168	0.9986
**4**	0.2714	0.9902	0.05545	0.7819	0.9967
**5**	0.1023	0.9999	0.00103	0.4992	0.9942
**6**	0.0986	0.9999	0.00180	0.4337	0.9965
Gentisein^d^	0.0535	0.9984	0.00011	0.3701	0.9974

All the tested compounds were examined in triplicates.

^a^ *K*_SV_ values means Stern-Volmer constant.

^b^ *K*_A_ means the affinity constants.

^c^ *n* means the binding number of xanthone to the BNA.

^d^ Gentisein was used as a positive control with the basic xanthone skeleton.

### 3.4 Predicting the binding modes between the new compounds and the protein by molecular docking

**FIGURE 6:**
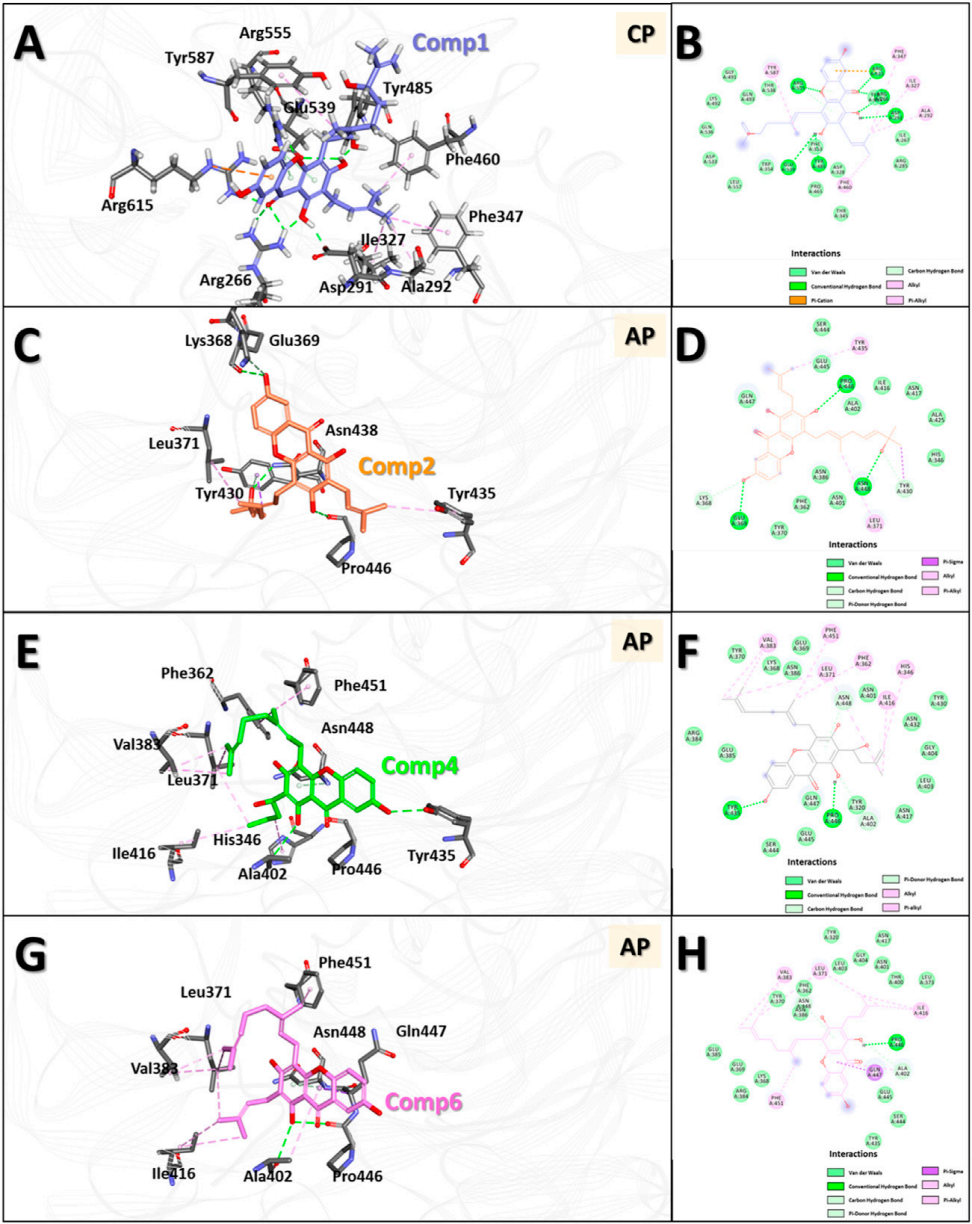
3D and 2D binding modes of the four compounds with the protein obtained by molecular docking (MD) studies. 3D and 2D binding modes and molecular interactions of compound **1 (A,B)**, **2 (C,D)**, **4 (E,F)** and **6 (G,H)** were displayed with the key residues in the binding site of the neuraminidase. In panel **(A,C,E,G)**, molecules are in ball-and-stick model to show clear intermolecular 3D interactions, while in panel **(B,D,F,H)**, in order to show entire interaction of the compound with the neighbor residues in the binding pocket of the protein, 2D molecular interaction diagrams were used. All the interactions are represented in dashed lines. The conventional hydrogen bonds are represented in green, carbon hydrogen bonds are shown in light green, π-sigma interactions are shown in purple, π-anion interactions are shown in orange, π-alkyl interactions are shown in pink, respectively.

Molecular docking (MD) was used to investigate the binding modes of compounds **1**, **2**, **4** and **6** at the *Clostridium perfringens* neuraminidase binding pocket. MD is considered a superior method for predicting the binding modes of given small molecules to the active site of proteins (Meng et al., 2011). Prior to the MD calculations, we collected information about the 3D structure of the protein and performed structural preparation for use in the study. Interestingly, the *Clostridium perfringens* neuraminidase target has two defined binding sites: the catalytic site and the allosteric site. In this study, based on the experimental observation, compound **1**, which was classified as a competitive inhibitor, was docked to the catalytic site, and the remaining compounds **2**, **4**, and **6** were docked to the allosteric site of the protein. The MD results showed that the docking scores of compounds **1**, **2**, **4**, and **6** were 72.68, 61.6, 67.3 and 61.29, respectively. The binding affinity of compound **1** was the best overall and for the allosteric site, that of compound **4** was the best, followed by **6**. The binding affinity of compound **2** was relatively low in comparison. Then, to optimize the binding structures, the best docking complexes of the four compounds were minimized using DS to minimize the energy. The resulting structures were analyzed for binding modes/intermolecular interactions and electrostatic surface models were generated (Figure 6).

The results showed that compound **1** had six conventional hydrogen bonds with Arg266, Asp291, Tyr485, Glu539, Arg555, and Arg615 and one carbon hydrogen bond with Arg555. In addition, compound **1** had five alkyl interactions with Ala292, Ile327, Phe347, Phe460, and Tyr587 and pi-cation interactions with Arg615. Several other residues also interacted with compound **1** via van der Waals interactions to anchor the compound to the binding pocket (Figures 6A, B). Compound **2** formed three conventional hydrogen bonds with Glu369, Pro446, and Asn448, and two carbon hydrogen bonds with Lys368 and Tyr430. Apart from this, two alkyl interactions with Leu371 and Tyr435, and a π-σ interaction with Tyr430 were also formed. In addition, several other residues interacted with compound **2** by van der Waals interactions to accommodate the compound in the binding pocket (Figures 6C, D). Compound **4** formed two conventional hydrogen bonds with Tyr435 and Pro446 and two carbon hydrogen bonds with Ala402 and Asn448. Six alkyl interactions were formed with His346, Phe362, Leu371, Val383, Ile416, and Phe451. Moreover, several other residues accommodated compound **4** in the binding pocket via van der Waals interactions (Figures 6E, F). Compound **6** formed one conventional hydrogen bond with Pro446 and two carbon hydrogen bonds with Ala402 and Asn448. Five alkyl interactions were formed with Leu371, Val383, Ala402, Ile416, and Phe451, and π-σ interactions were formed with Gln447. Several other residues also accommodated the compound via van der Waals interactions (Figures 6G, H).

In the interaction of compound **1** with the catalytic site in the overall binding pattern, the gentisein moiety undergoes interactions with various residues outside the pocket, and the geranyl and prenyl groups also undergo strong interactions with the interior of the pocket. These interactions are the driving force behind the binding stability (Figures 6A, B). The binding mode data showed that the interaction of compounds **4** and **6** with the binding pocket was more rigorous compared to that of compound **2**. The former two compounds interacted with the interior of the pocket via the geranyl and prenyl groups linked to gentisein to form a stable structure, whereas compound **2** could not engage in the interaction to the same extent. These results are in good agreement with our experimental observation, which indicated that the activities of compound **4** and **6** were much higher than that of compound **2**.

### 3.5 Binding mode comparison of compound 2, 4, and 6 using the MDS structures

**FIGURE 7:**
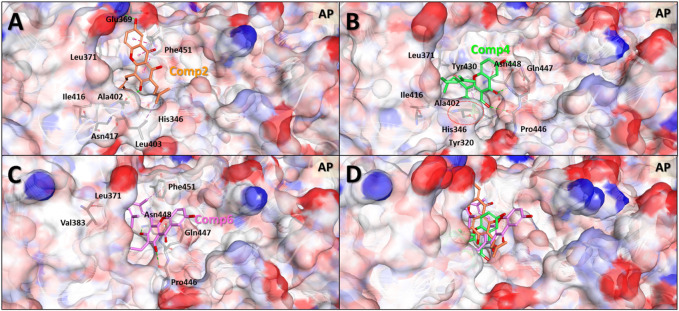
Predicted binding modes based on molecular dynamics simulation structures of the three compounds in the allosteric pocket of *Clostridium perfringens* neuraminidase. Binding mode and molecular interactions of compounds **2 (A)**, **4 (B)**, **6 (C)** and **4** and **6** together **(D)** of the three non-competitive compounds were shown with the key residues of the allosteric binding site of the neuraminidase. Each panel images used ball and stick model for the ligand to show the interaction details and electrostatic surface model to show the binding pocket geometry of the protein. The red dotted circle in panel **(B)** indicates the interaction part of hydroxyl (-OH) group of the molecule which is the only one difference between compounds **4** and **6**.

In general, the MDS approach can provide structural information of the molecules that is much more accurate compared to the MD results. This is because, contrary to MD, MDS is able to consider the changes induced in the protein structure by the ligand binding. MDS calculations were therefore carried out on the protein-ligand complex of the selected compounds to investigate in greater detail the binding modes of compounds **2**, **4**, and **6** to the allosteric pocket. After 5 ns simulations, the stable protein-ligand complex structures were obtained and used for the analyses of the binding modes and intermolecular interactions (Figure 7 and Supplementary Figure S36).

The MDS binding mode data clearly showed that the more active compounds **4** and **6** interacted more solidly with the binding pocket than compound **2**. Figure 7 shows that the two molecules to a greater extent engage in binding inside the pocket than compound **2**. In terms of the overall shape, compounds **4** and **6** interacted with the geranyl and prenyl groups linked to gentisein and thus formed stable interactions with the interior of the pocket, whereas the compound **2** was unable to. These results correspond well with our early experimental observation that compounds **4** and **6** have higher activities than compound **2**. In addition, the difference in the activity between compounds **4** and **6** was also investigated using these MDS structures. These results showed that the hydroxyl group of the prenyl group in compound **4** formed a hydrogen bond with Ala402 of the protein and this interaction seemingly allowed the ligand to be located very deeply inside the pocket. This, in turn, appeared to stabilize the binding to compound **4** preferably to compound **6**. We consider this interaction to be the key difference between compounds **4** and **6**.

## 4 Conclusion

We succeeded in carrying out the first in-depth analysis of xanthones bearing geranyl and prenyl group from the roots of *C. cochinchinense*. Isolation yielded six xanthones, three of which proved to be new compounds (**1**–**3**). All these xanthones displayed significant inhibition against bacterial neuraminidase with an IC_50_ range of 0.38–38.9 µM. Subtle changes to the geranyl or prenyl group affected the inhibitory potency and mechanism, for example, **1** (IC_50_ = 0.38 µM, a competitive inhibitor) vs. **6** (IC_50_ = 9.8 µM, a non-competitive inhibitor). The most potent inhibitor **1** was demonstrated to engage in simple reversible slow binding behavior with a strong binding affinity (*K*_SV_ = 1.5792 × 10^5^ L·mol^−1^, *K*_A_ = 0.2443 × 10^6^ L·mol^−1^, and *n* = 1.2897). The results of the MD and MDS studies were in agreement with those of the *in vitro* enzyme studies. The MD and MDS studies also provided critical information regarding the role of the geranyl and prenyl groups to undergo subtle interaction with the interior of the pocket for stronger inhibition.

## Data Availability

The datasets presented in this study can be found in online repositories. The names of the repository/repositories and accession number(s) can be found in the article/Supplementary Material.
