# Supplementary material for: Investigation of bacterial neuraminidase inhibition of xanthones bearing geranyl and prenyl groups from Cratoxylum cochinchinense
Source: Front Chem. 2023 Aug 9;11:1245071. doi: 10.3389/fchem.2023.1245071 (PMC10445491; doi:10.3389/fchem.2023.1245071)
Supplement: Supplementary file 1 [file DataSheet1.pdf]

Supplementary information

Investigation of Bacterial Neuraminidase Inhibition of Xanthones Bearing Geranyl and Prenyl Groups from *Cratoxylum cochinchinense*

Jeong Yoon Kim[a,1], Zuo Peng Li[b,1], Gihwan Lee[c], Jeong Ho Kim[c], Abdul Bari Shah[c], Yong Hyun Lee[c], Ki Hun Park[c,*]

[a]Department of Pharmaceutical Engineering, IALS, Gyeongsang National University, Jinju, 52725, Republic of Korea

[b]*State Key Laboratory Basis of Xinjiang Indigenous Medicinal Plants Resource Utilization, Xinjiang Technical Institute of Physics and Chemistry, Chinese Academy of Sciences, Urumqi 830011, People's Republic of China*

[c]Division of Applied Life Science (BK21 Four), IALS, Gyeongsang National University, Jinju, 52828, Republic of Korea

List of supporting data

- Figures 1-6: NMR and HREIMS data of new xanthone **1**

- Figures 7-12: NMR and HREIMS data of new xanthone **2**

- Figures 13-18: NMR and HREIMS data of new xanthone **3**

- Figures 19-21: IR spectra of new xanthones **1**-**3**

- Figures 22-24: UV spectra of new xanthones **1**-**3**

- Figures 25-33: NMR spectra of xanthones **4**-**6**

- Figure 34: Lineweaver Burk plots of xanthones **2**, **3**, **5**, and **6**

- Figure 35: Dixon plots of xanthones **2**, **3**, **5**, and **6**

- Figure 36: Fluorescence quenching effect of xanthones **2**, **6**, and gentisein

- Figure 37: Fluorescence residues of neuraminidase from *Clostridium perfringens*

- Figure 38. Predicted binding modes based on molecular docking (MD) structures between the four compounds and the *Clostridium perfringens* neuraminidase

- Figure 39. 3D and 2D binding modes of the three compounds in the allosteric pocket of the protein obtained by molecular dynamics simulation (MDS) studies

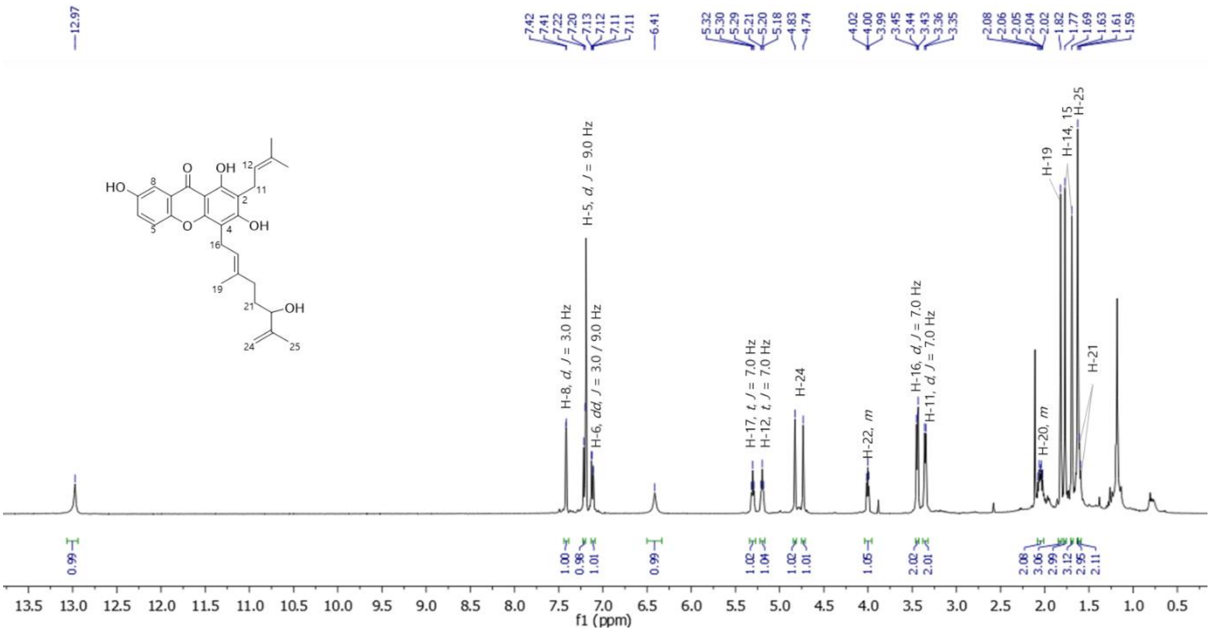

Figure S1. ^{1}H-NMR spectrum of xanthone **1** (500 MHz, Acetone-d_6)

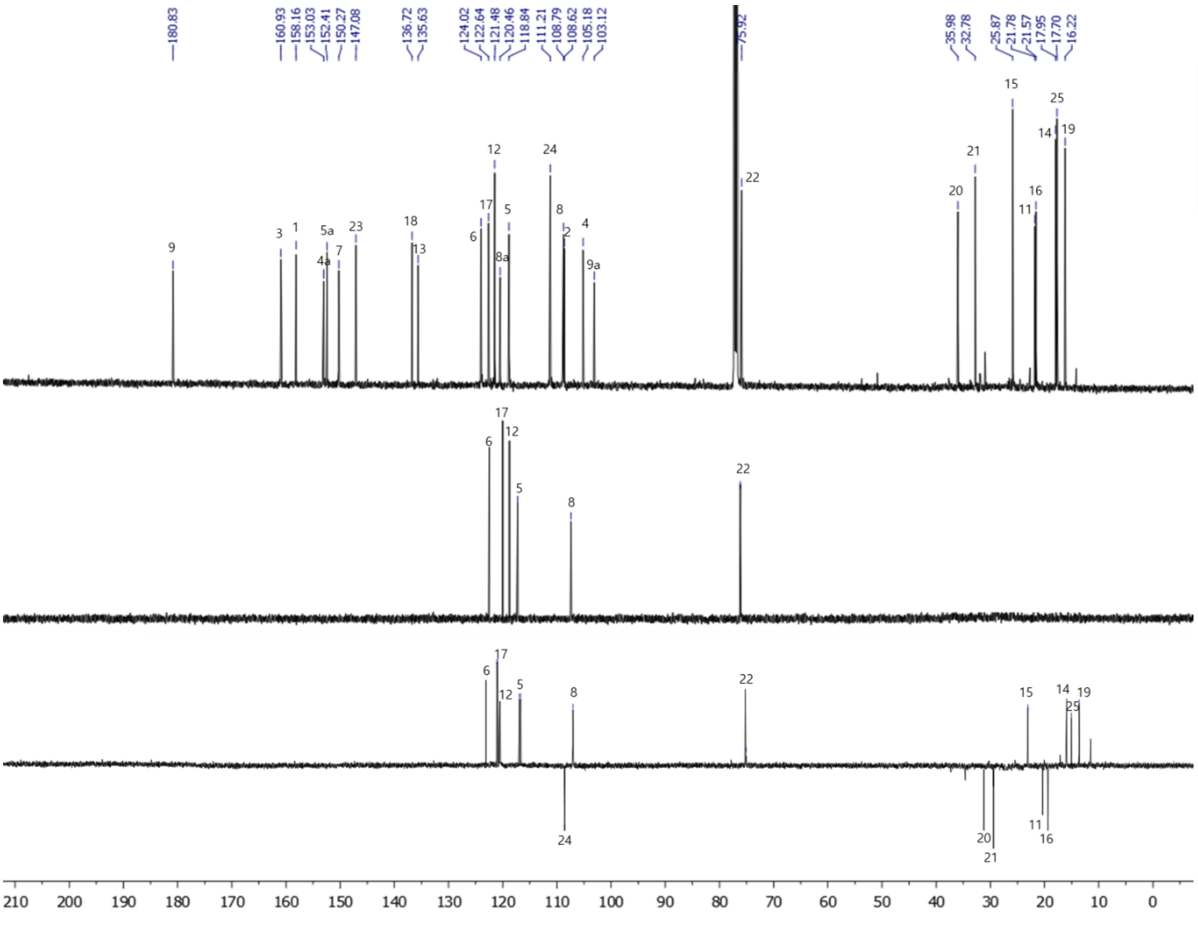

Figure S2. ^{13}C-NMR, DEPT 90, and 135 spectra of xanthone **1** (125 MHz, Acetone-d_6)

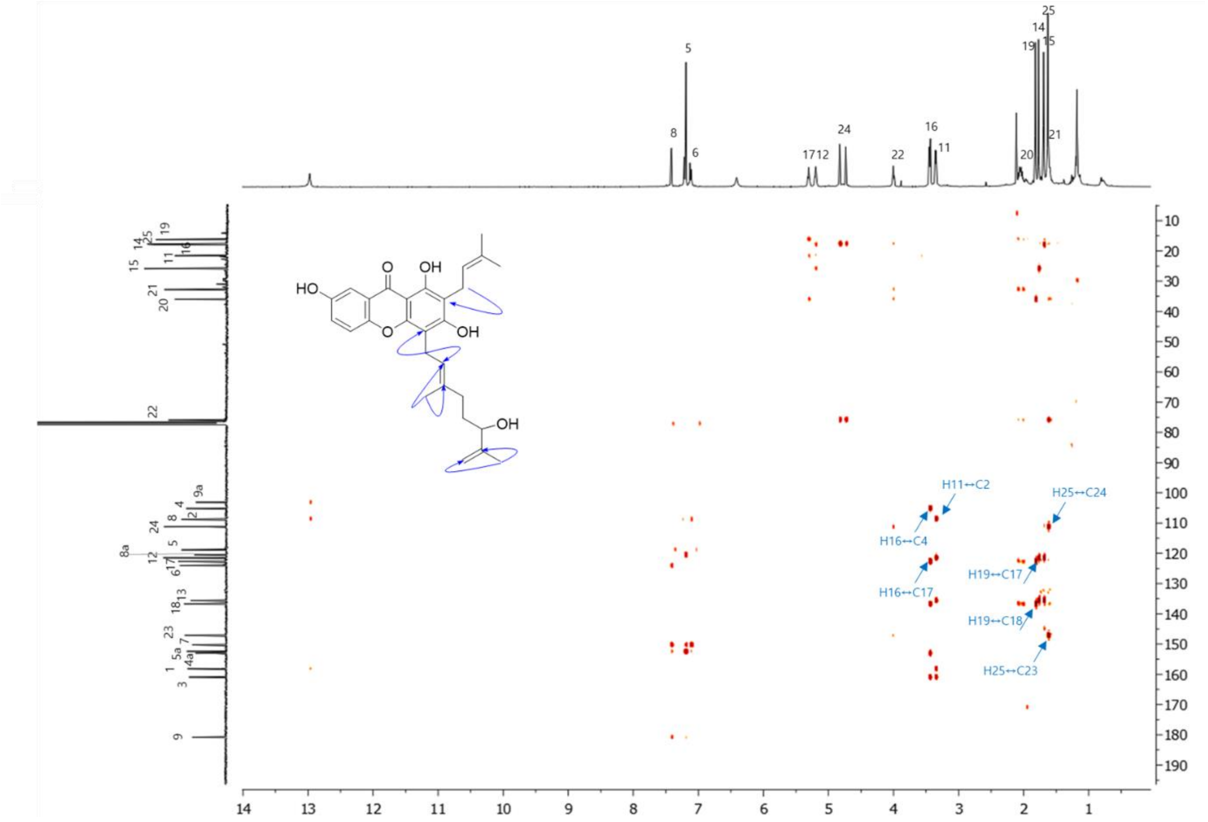

Figure S3. HMBC spectra of xanthone **1** (500 MHz, Acetone-d_6)

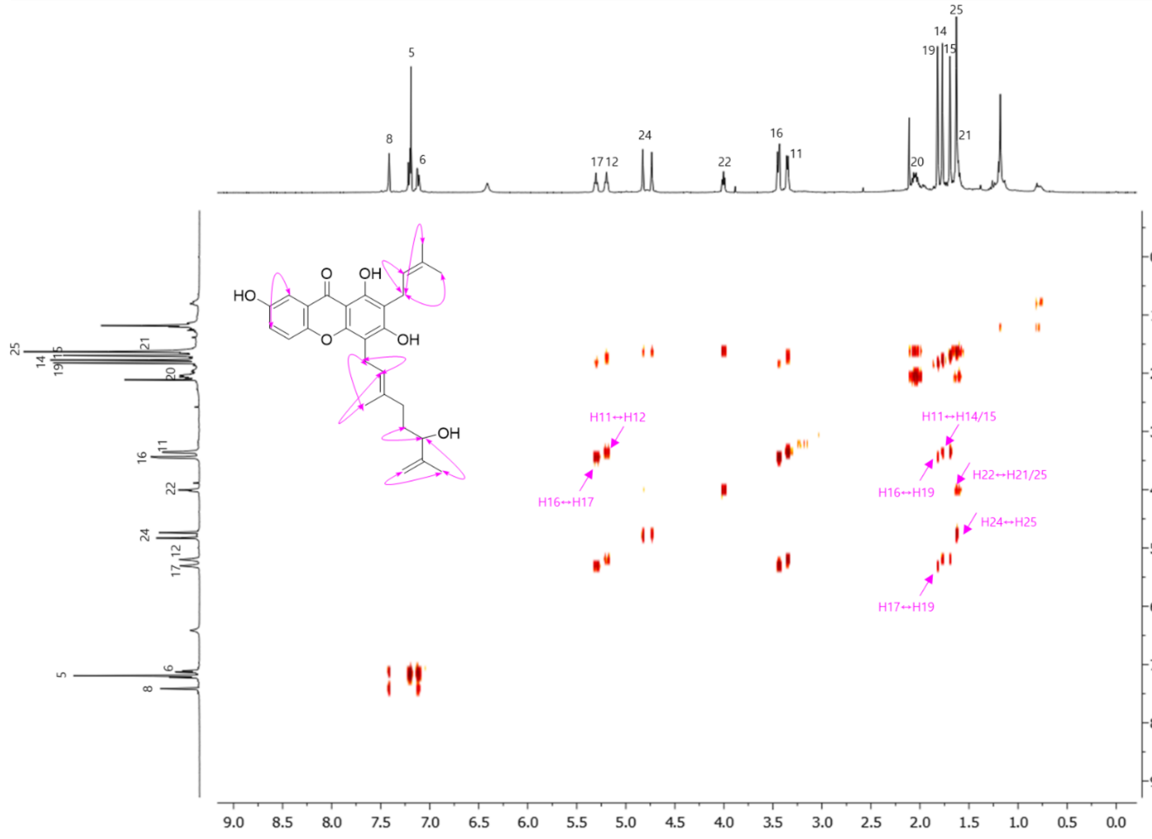

Figure S4. COSY spectra of xanthone **1** (500 MHz, Acetone-d_6)

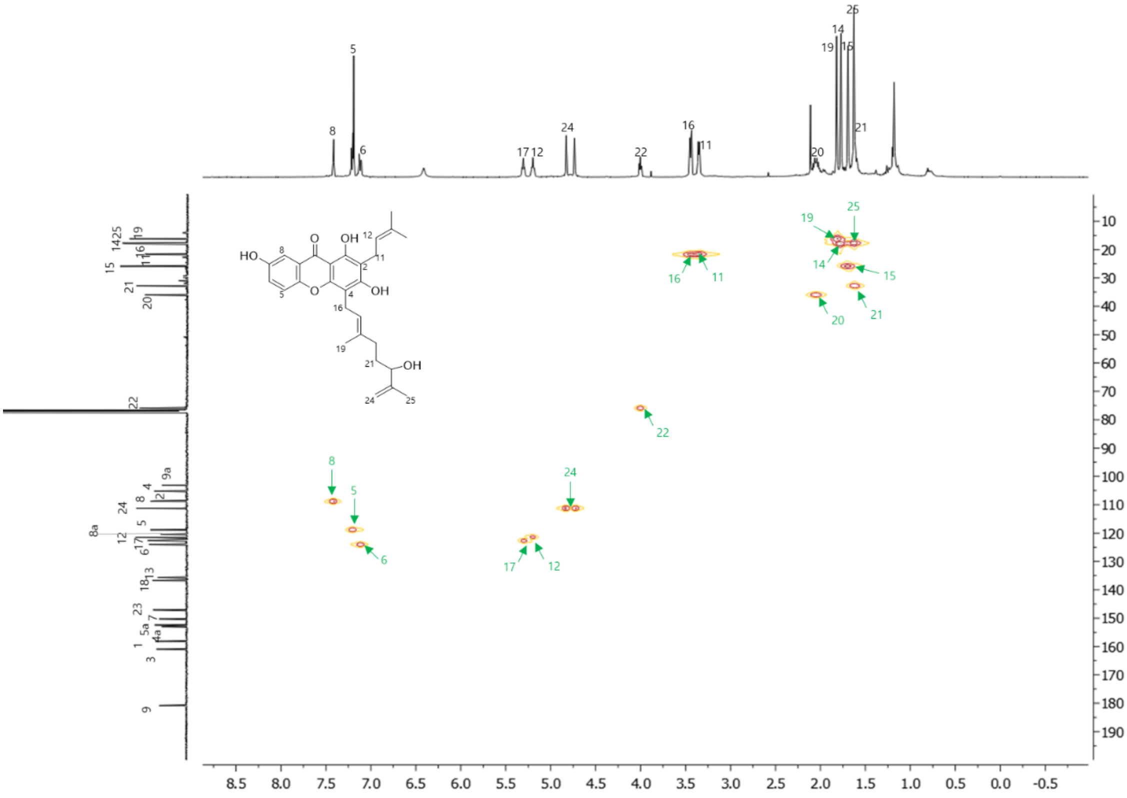

Figure S5. HMQC spectra of xanthone **1** (500 MHz, Acetone-d_6)

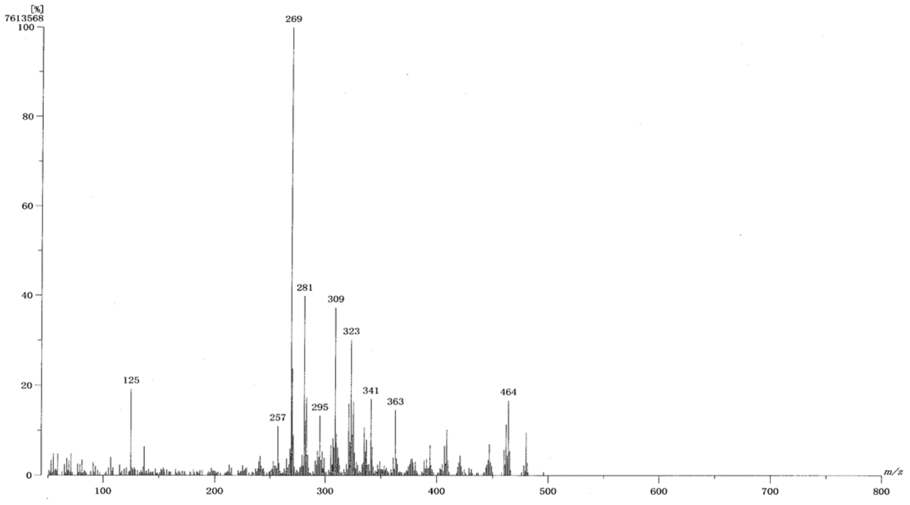

Instrument : MStation
Sample : –
Note : –
Inlet : Direct   Ion Mode : EI+
RT : 0.90 min   Scan# : 19
Elements : C 100/1, H 100/1, O 10/1
Mass Tolerance   : 1000ppm, 3mmu if m/z > 3
Unsaturation (U.S.) : -0.5 – 20.0

	Observed m/z	Int%	Err[ppm / mmu]	U.S.	Composition
1	464.2197	100.00	-0.4 / -0.2	13.0	C28 H32 O6

Figure S6. HREIMS data of xanthone **1**

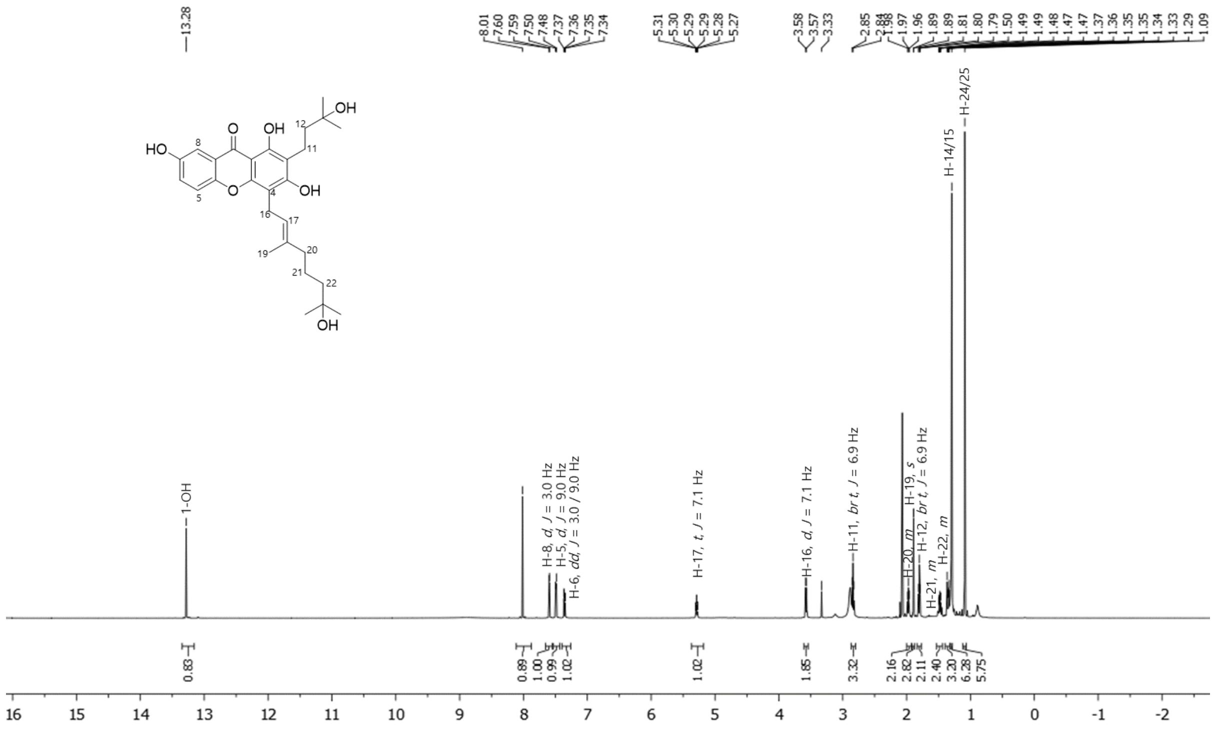

Figure S7. ^{1}H-NMR spectrum of xanthone **2** (500 MHz, Acetone-d_6)

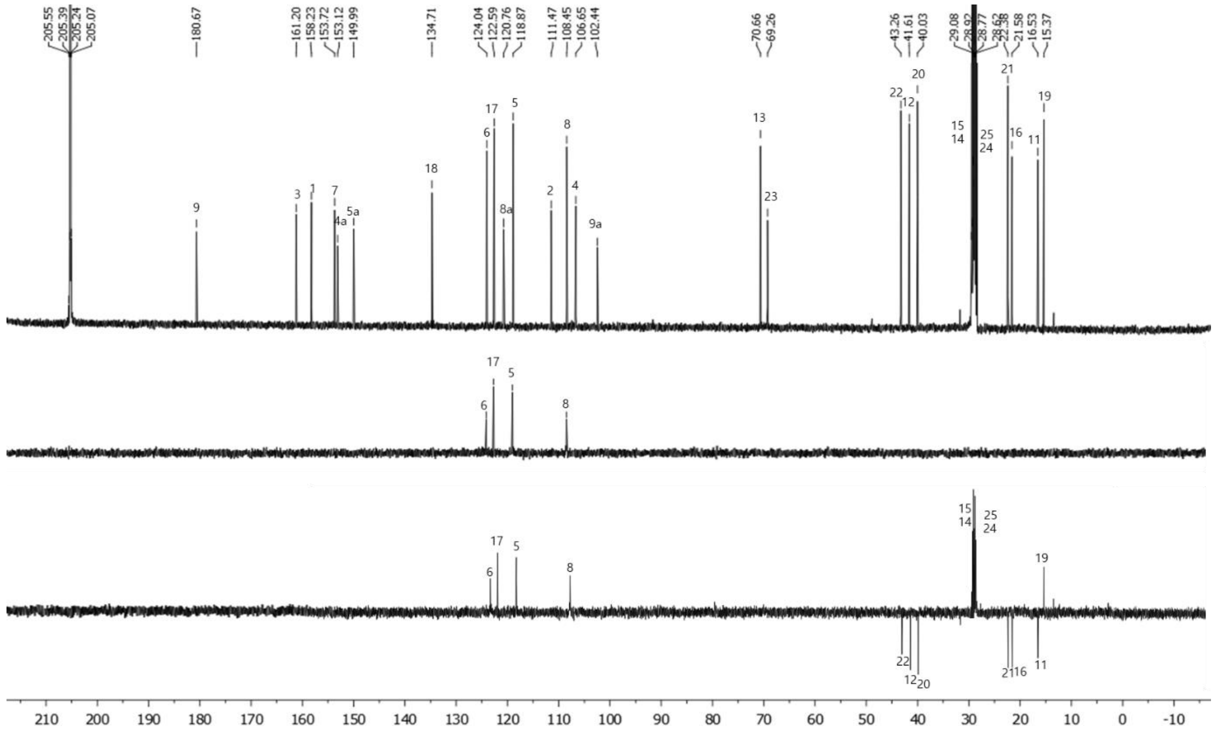

Figure S8. ^{13}C-NMR, DEPT 90, and 135 spectra of xanthone **2** (125 MHz, Acetone-d_6)

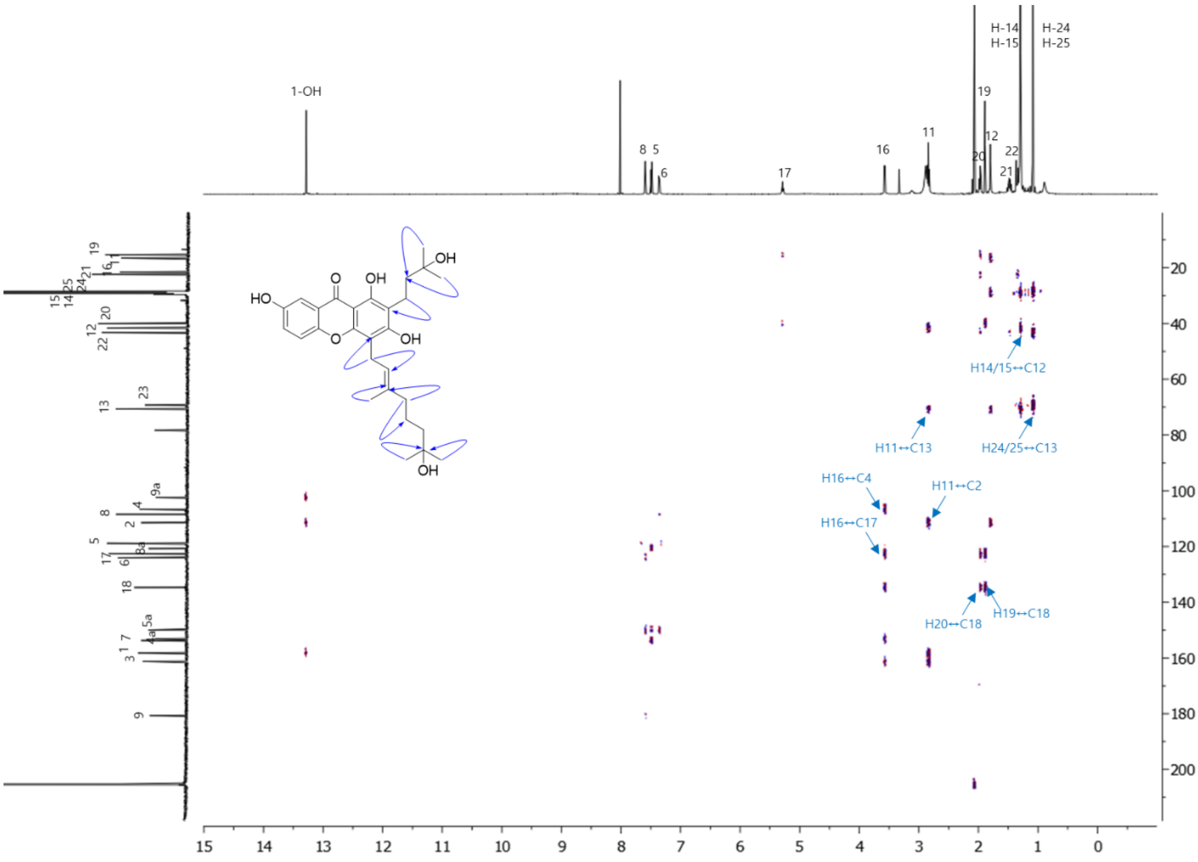

Figure S9. HMBC spectra of xanthone **2** (500 MHz, Acetone-d_6)

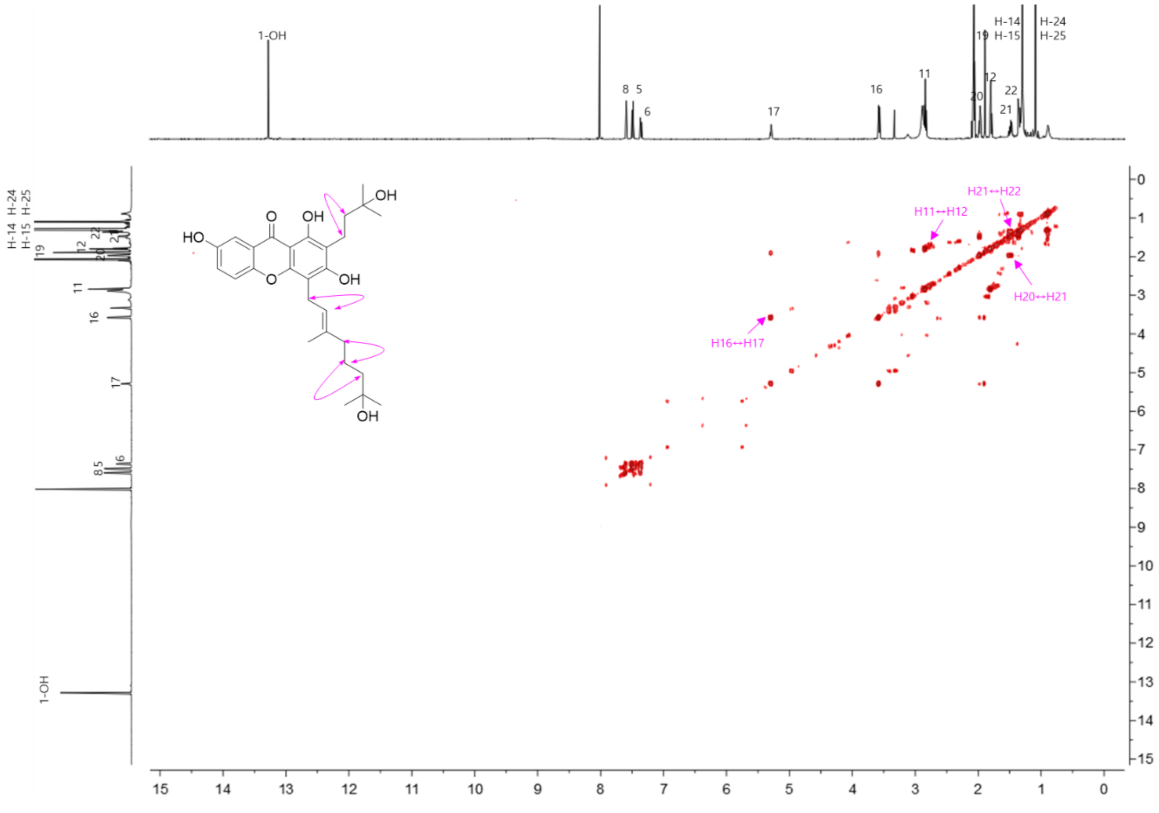

Figure S10. COSY spectra of xanthone **2** (500 MHz, Acetone-d_6)

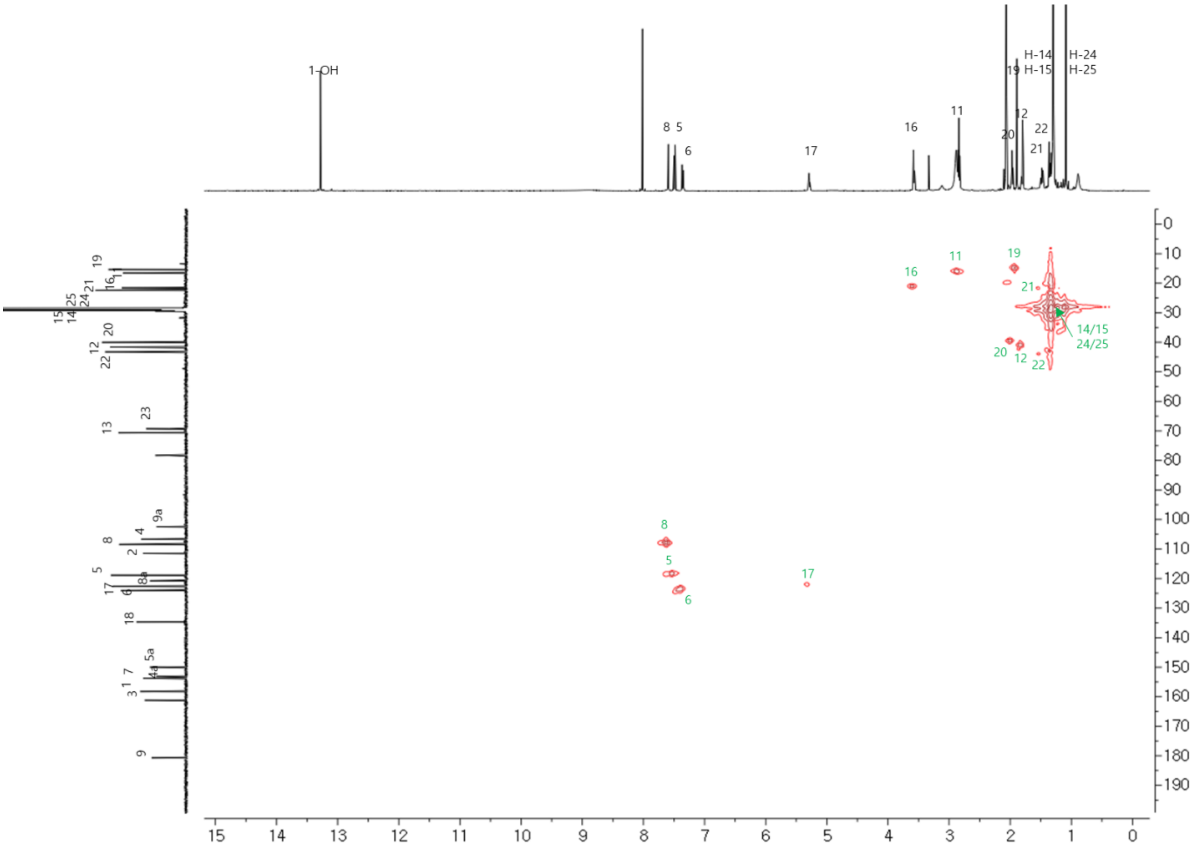

Figure S11. HMQC spectra of xanthone **2** (500 MHz, Acetone-d_6)

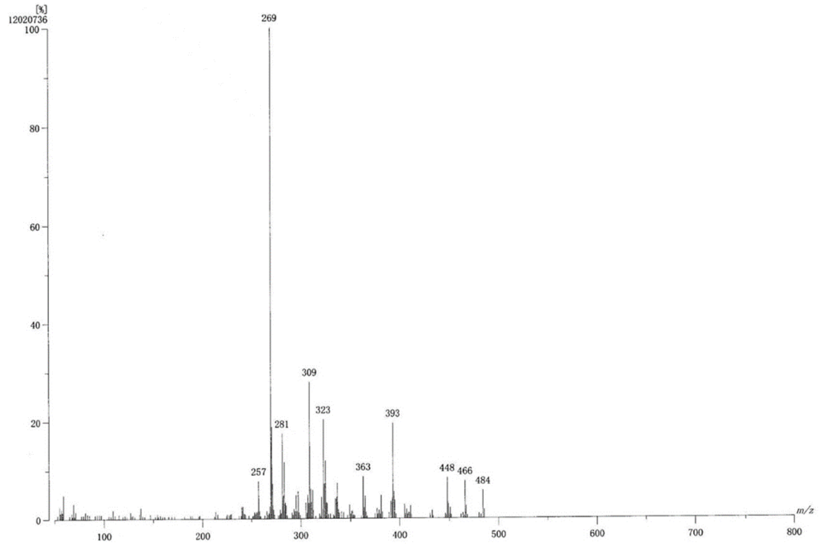

Instrument : MStation
Sample : –
Note : –
Inlet : Direct Ion Mode : EI+
RT : 0.57 min Scan# : 18
Elements : C 100/1, H 100/1, O 10/1
Mass Tolerance : 1000ppm, 3mmu if m/z > 3
Unsaturation (U.S.) : -0.5 – 20.0

	Observed m/z	Int%	Err[ppm / mmu]	U.S.	Composition
1	484.2461	35.63	-0.0 / -0.0	11.0	C28 H36 O7

Figure S12. HREIMS data of xanthone **2**

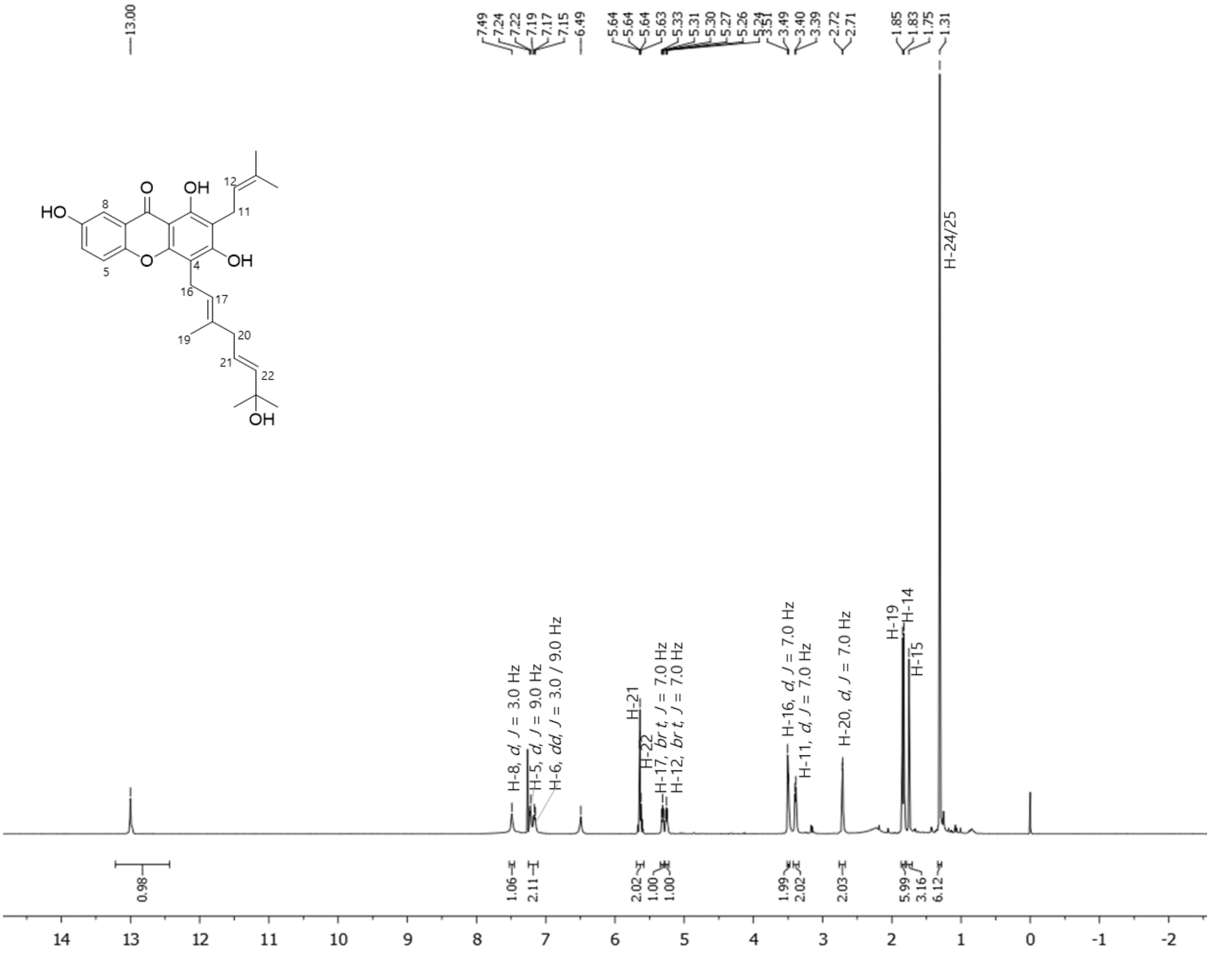

Figure S13. ^{1}H-NMR spectrum of xanthone **3** (500 MHz, $CDCl_3$)

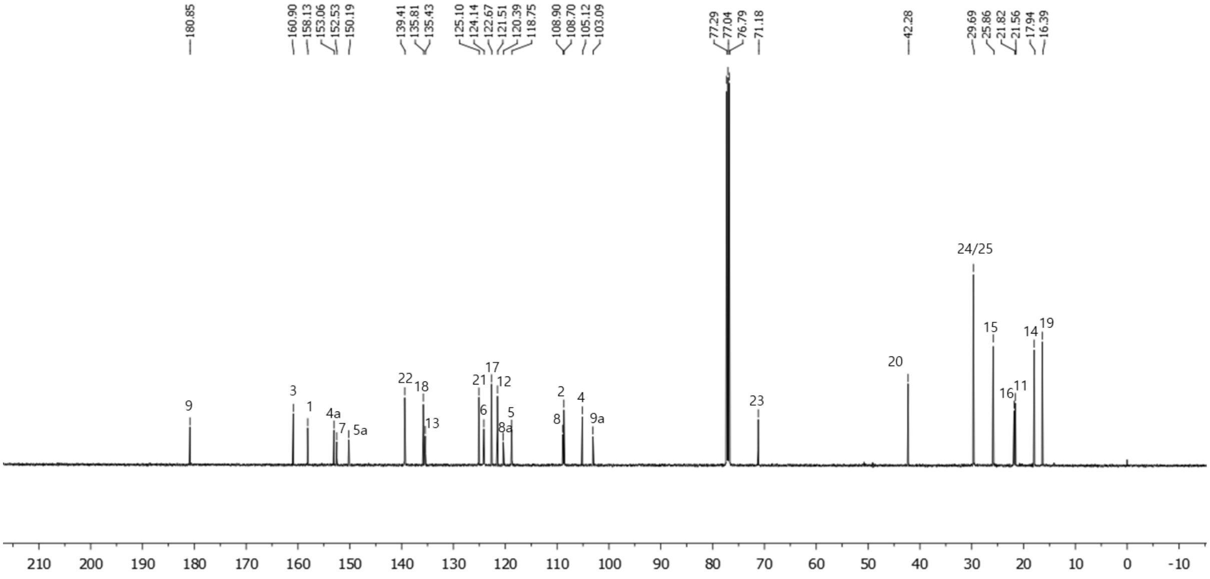

Figure S14. ^{13}C-NMR of xanthone **3** (125 MHz, $CDCl_3$)

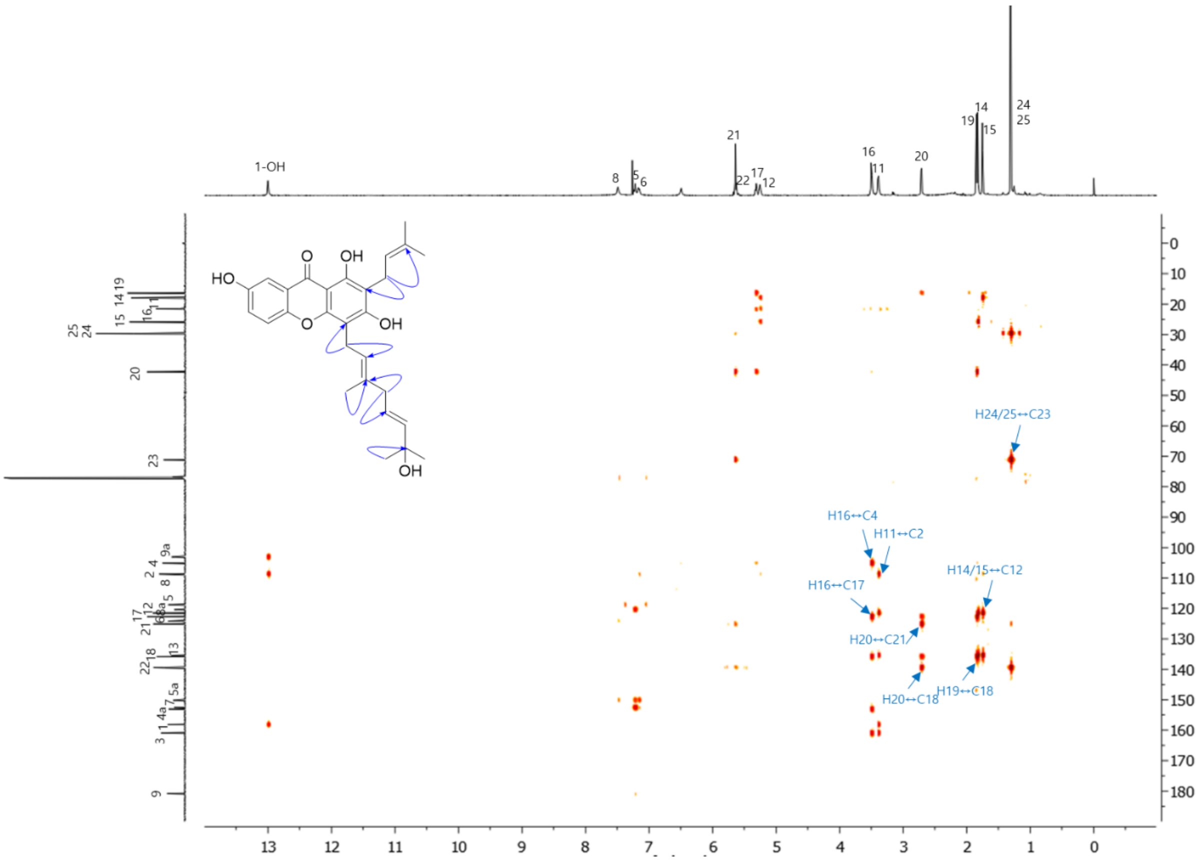

Figure S15. HMBC spectra of xanthone **3** (500 MHz, $CDCl_3$)

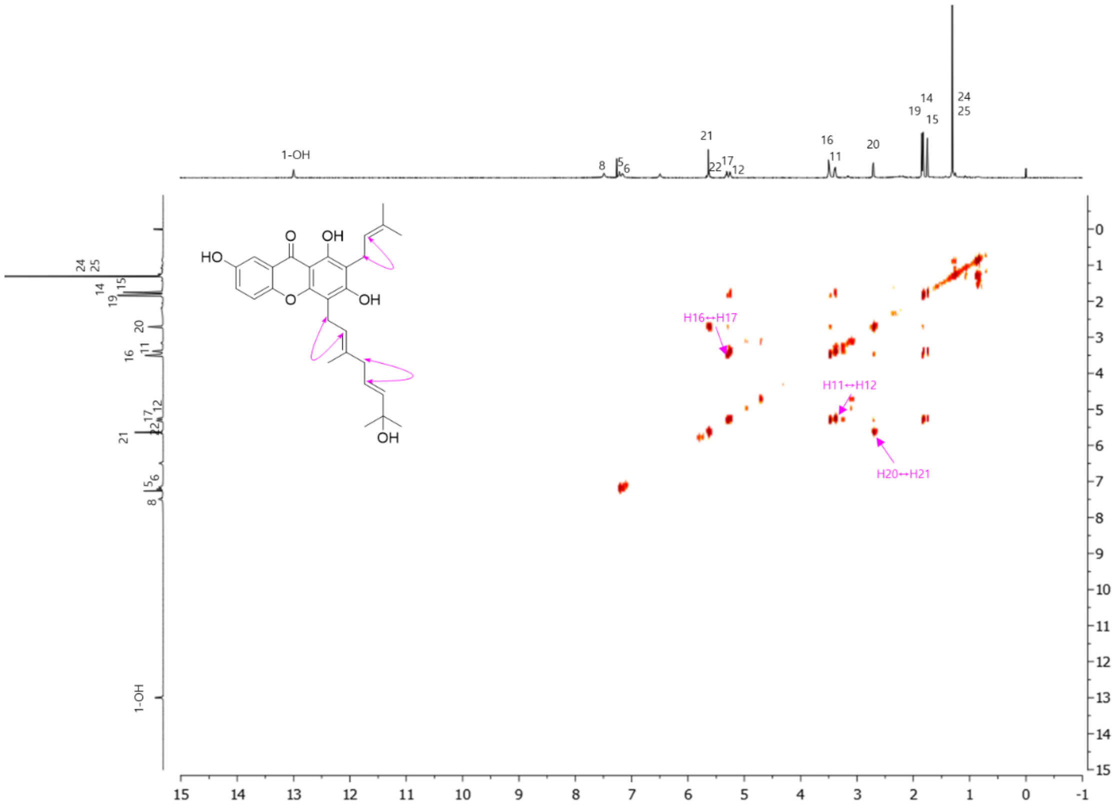

Figure S16. COSY spectra of xanthone **3** (500 MHz, $CDCl_3$)

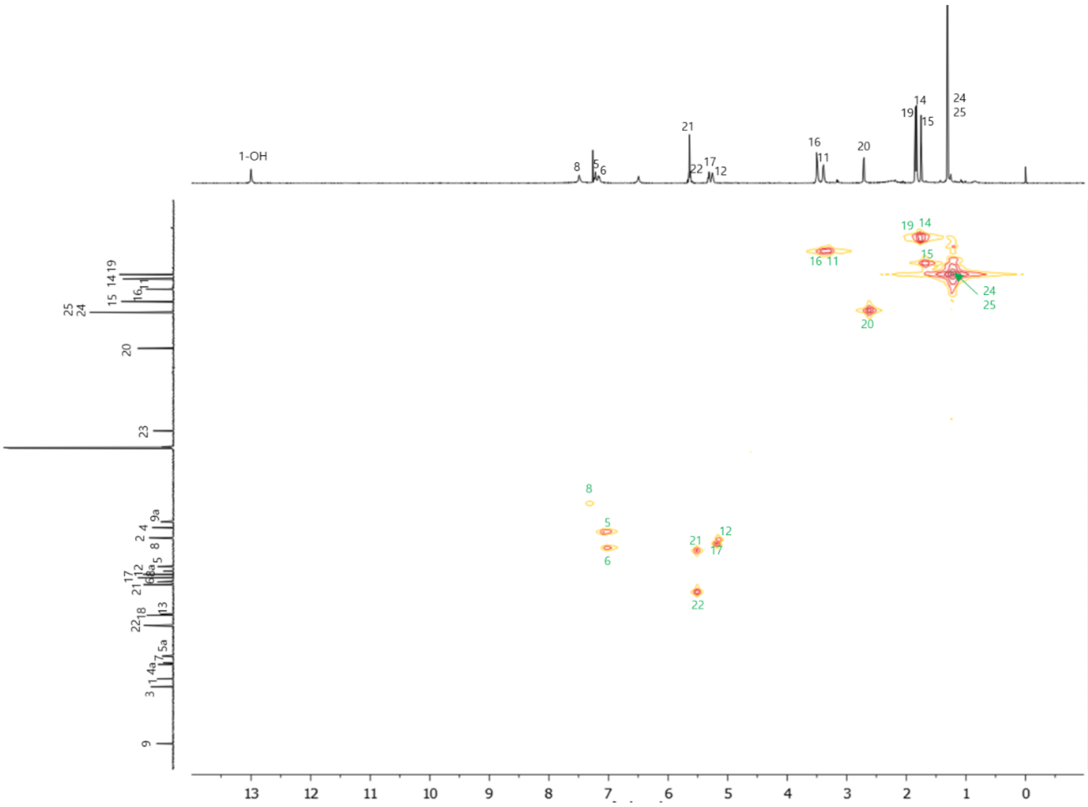

Figure S17. HMQC spectra of xanthone **3** (500 MHz, $CDCl_3$)

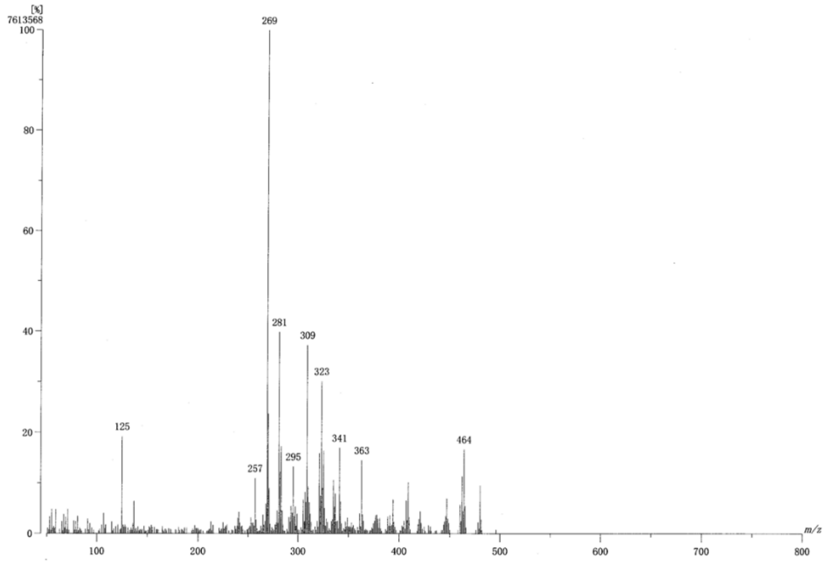

Instrument : MStation
Sample : -
Note : -
Inlet : Direct Ion Mode : EI+
RT : 0.90 min Scan# : 19
Elements : C 100/1, H 100/1, O 10/1
Mass Tolerance : 1000ppm, 3mmu if m/z > 3
Unsaturation (U.S.) : -0.5 - 20.0

	Observed m/z	Int%	Err[ppm / mmu]	U.S.	Composition
1	464.2197	100.00	-0.4 / -0.2	13.0	C28 H32 O6

Figure S18. HREIMS data of xanthone **3**

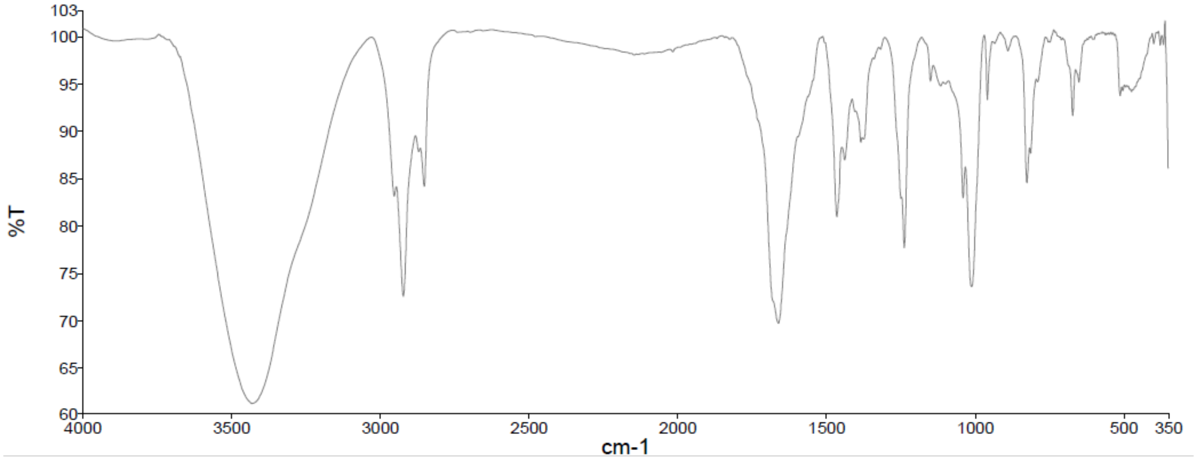

Figure S19. IR spectra of xanthone **1**

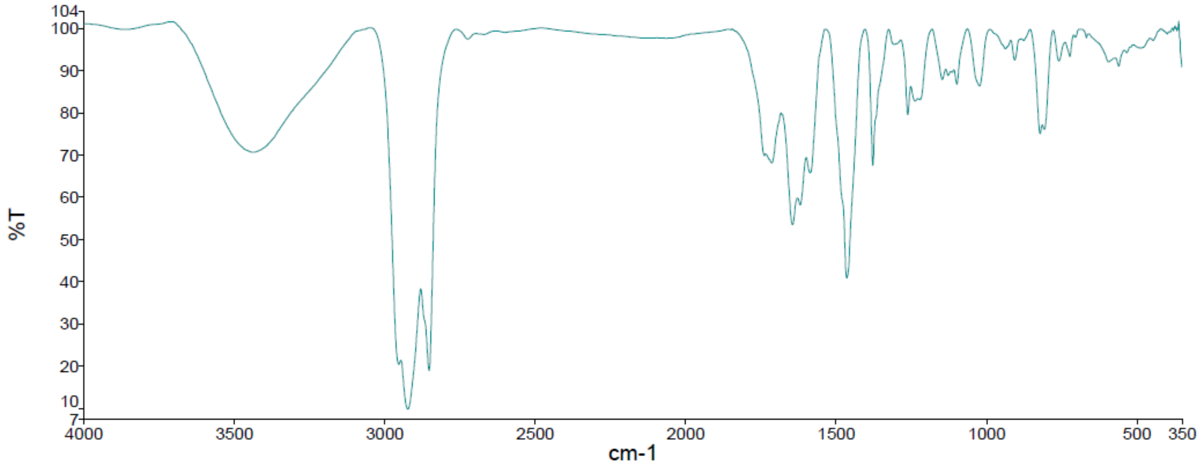

Figure S20. IR spectra of xanthone **2**

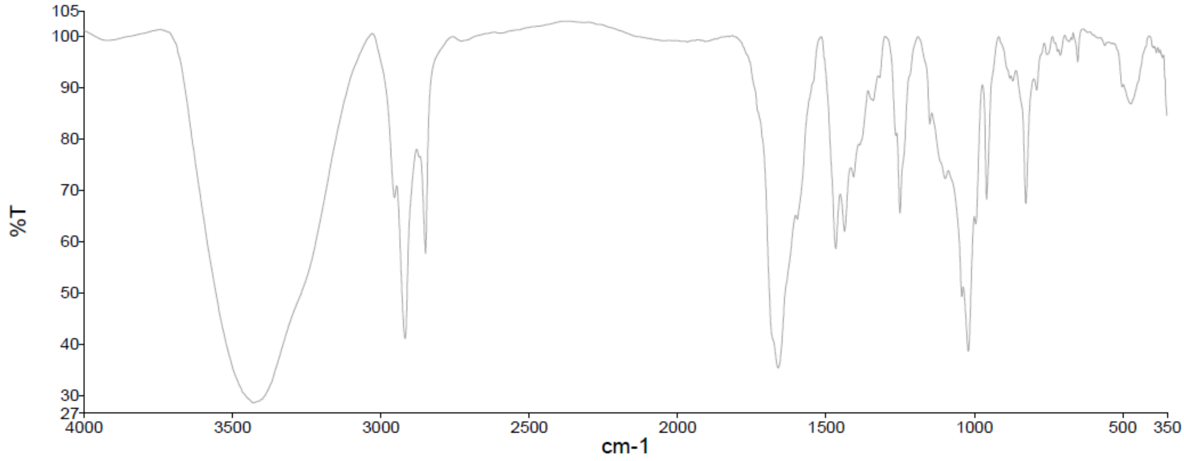

Figure S21. IR spectra of xanthone **3**

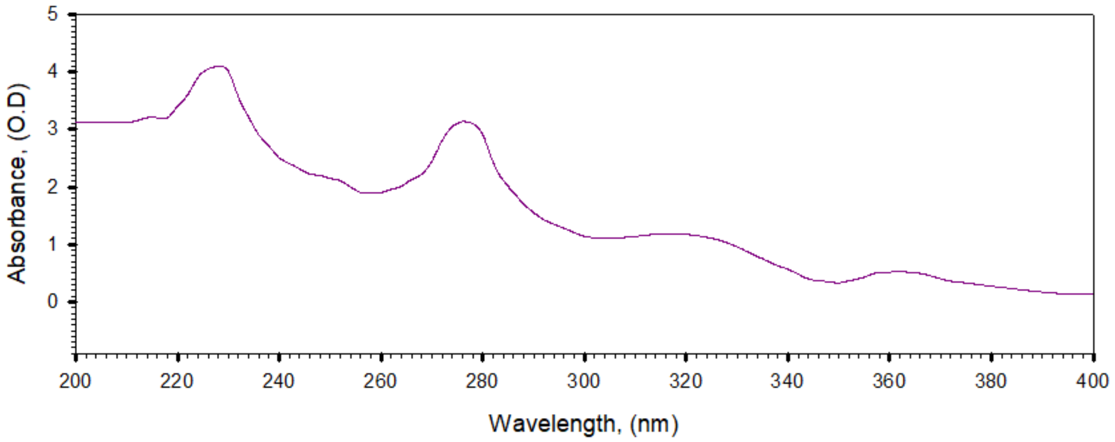

Figure S22. UV spectra of xanthone **1**

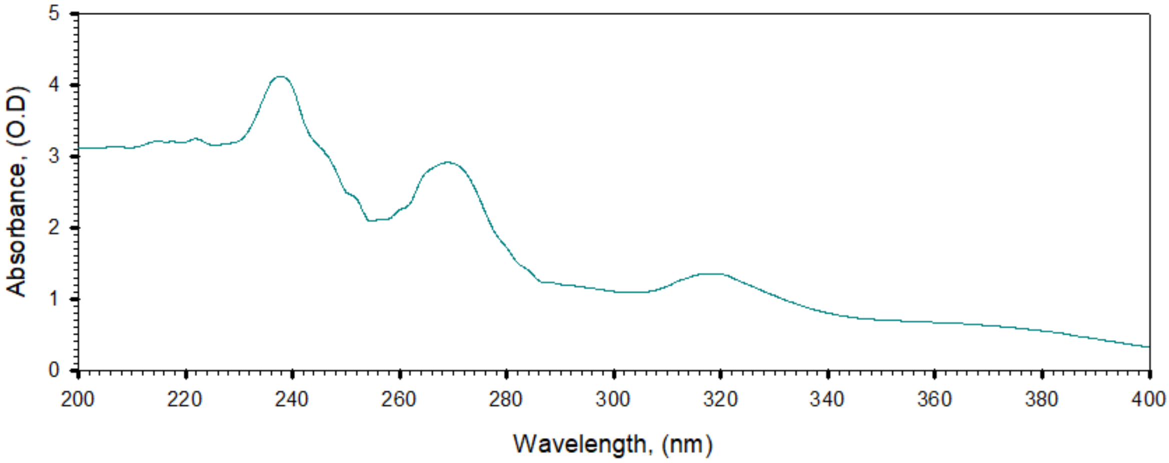

Figure S23. UV spectra of xanthone **2**

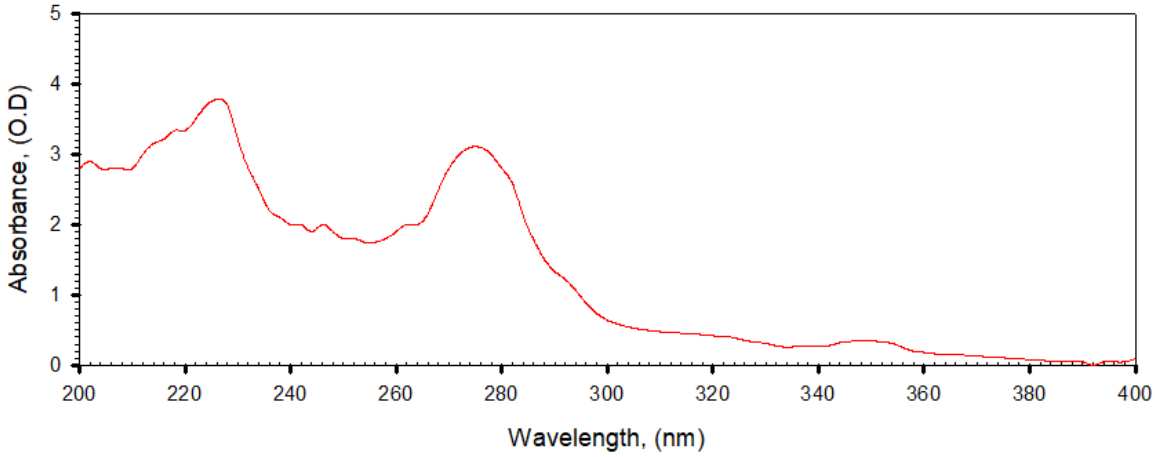

Figure S24. UV spectra of xanthone **3**

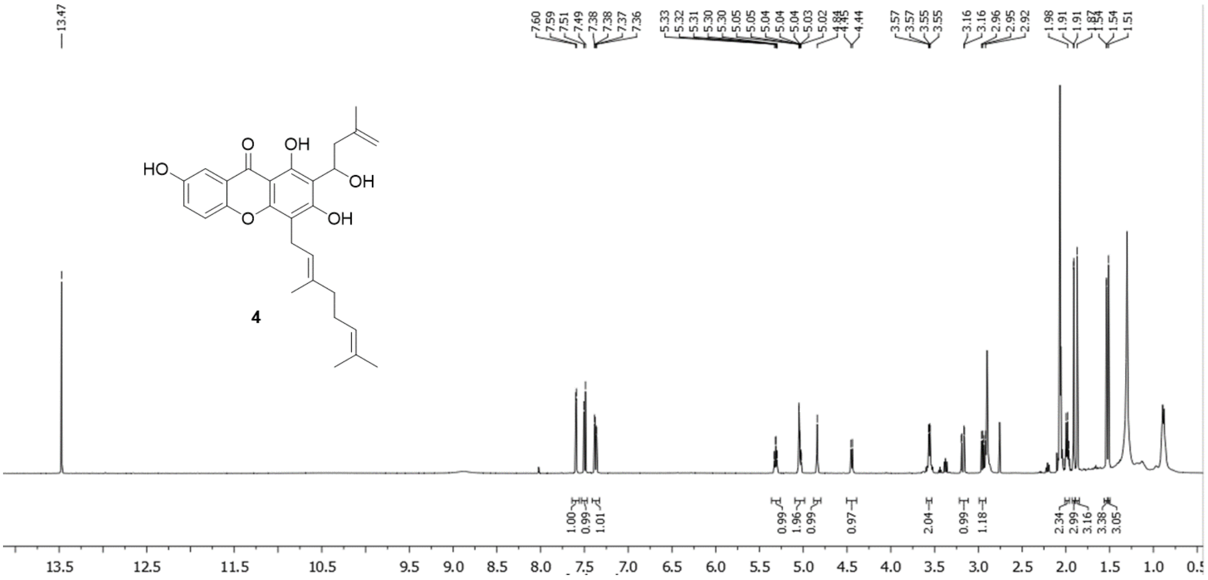

Figure S25. ^{1}H-NMR spectrum of xanthone **4** (500 MHz, Acetone-d_6)

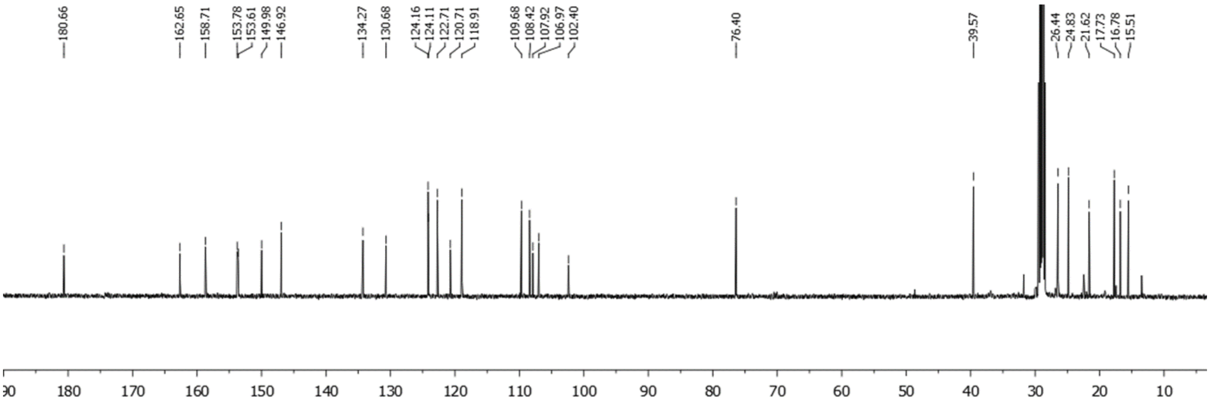

Figure S26. ^{13}C-NMR of xanthone **4** (125 MHz, Acetone-d_6)

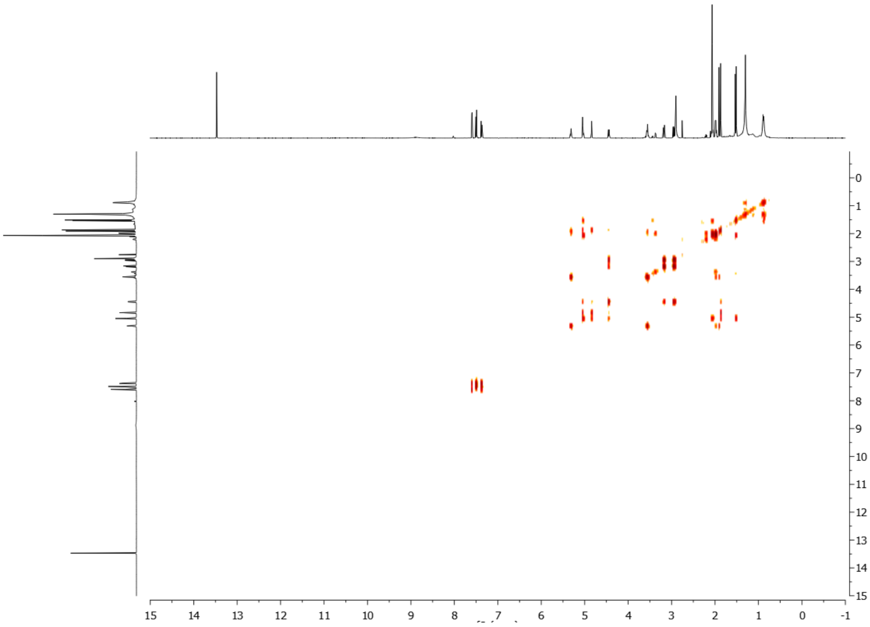

Figure S27. HMBC spectra of xanthone **4** (500 MHz, Acetone-d_6)

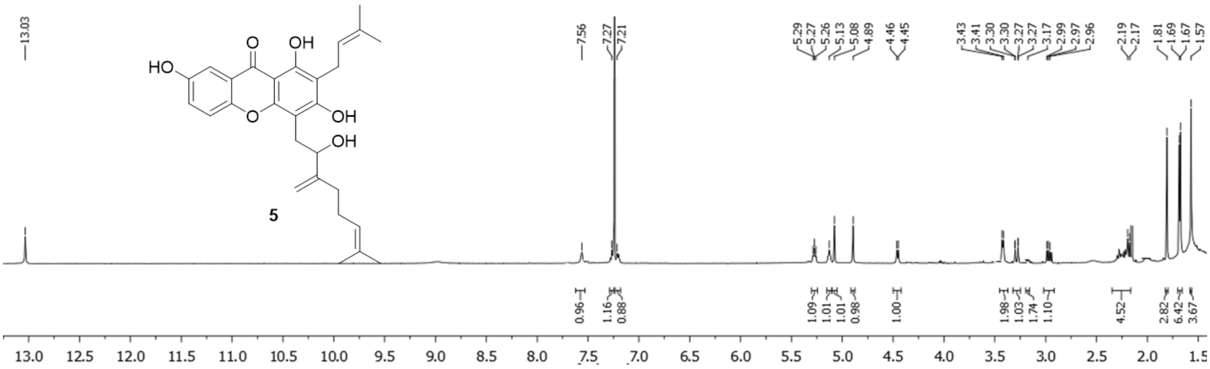

Figure S28. ^{1}H-NMR spectrum of xanthone **5** (500 MHz, $CDCl_3$)

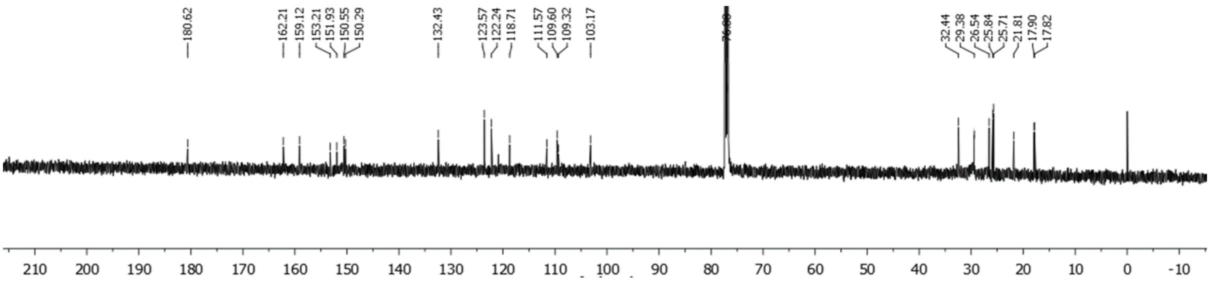

Figure S29. ^{13}C-NMR of xanthone **5** (125 MHz, $CDCl_3$)

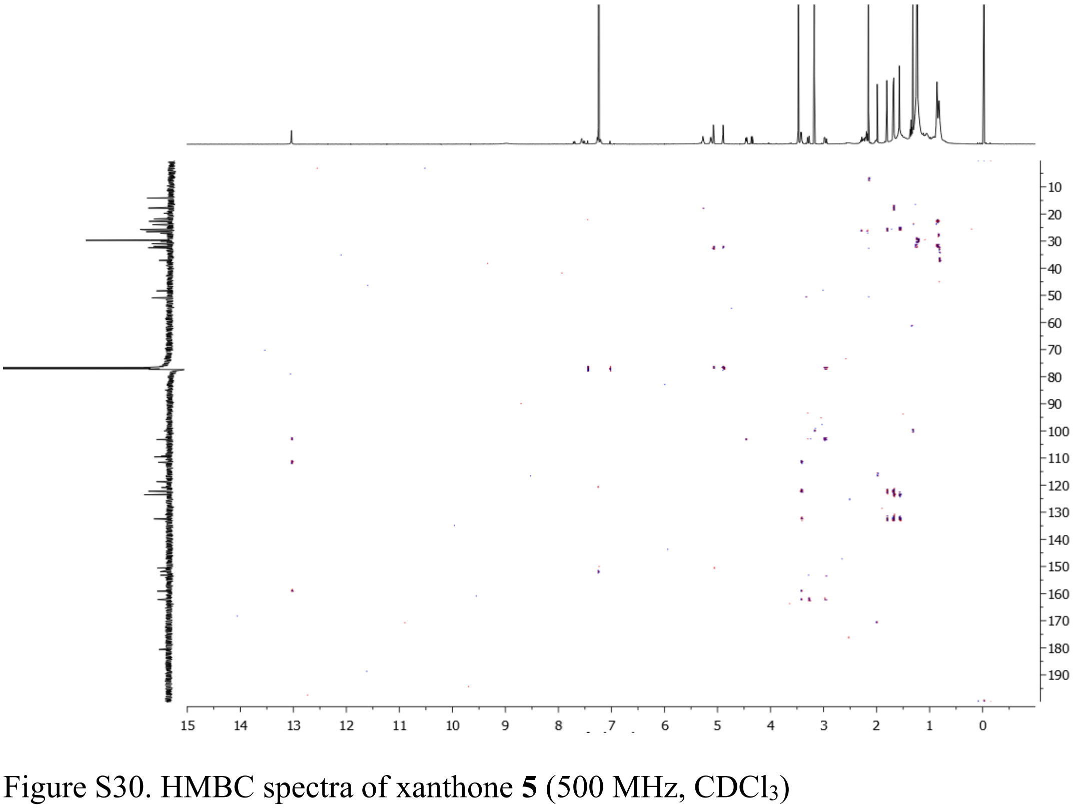

Figure S30. HMBC spectra of xanthone **5** (500 MHz, $CDCl_3$)

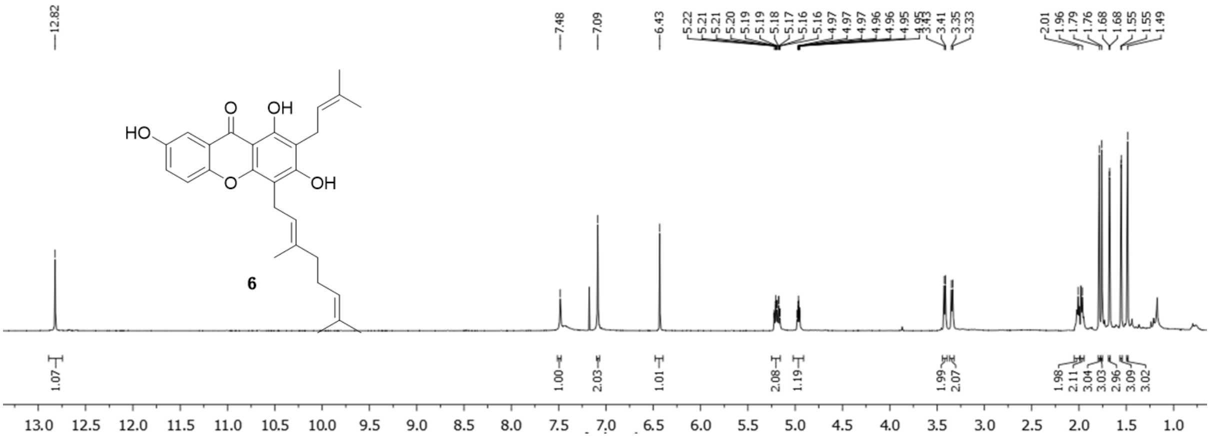

Figure S31. ^{1}H-NMR spectrum of xanthone **6** (500 MHz, $CDCl_3$)

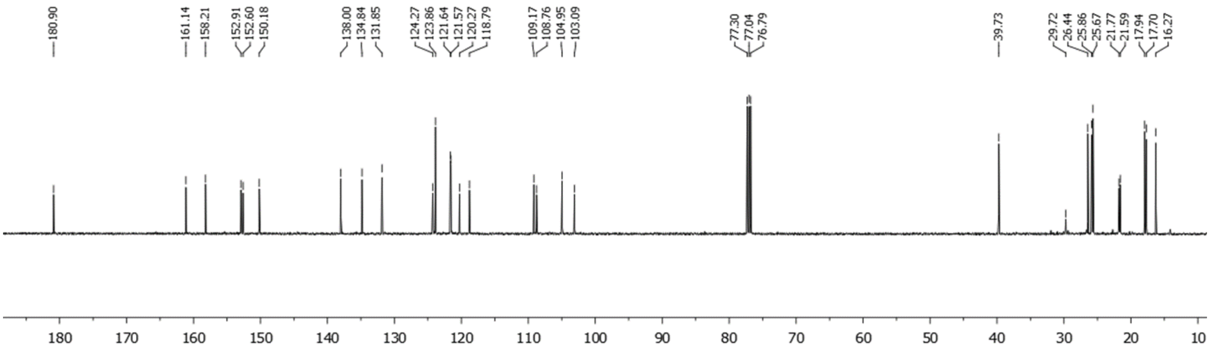

Figure S32. ^{13}C-NMR of xanthone **6** (125 MHz, $CDCl_3$)

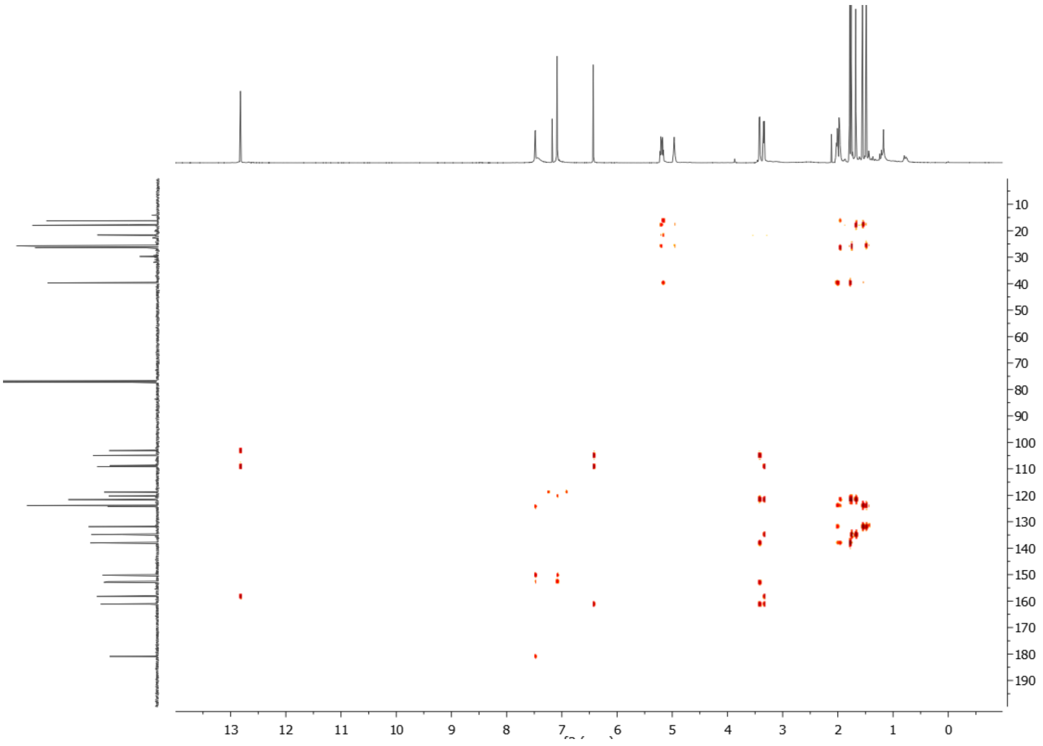

Figure S33. HMBC spectra of xanthone **6** (500 MHz, $CDCl_3$)

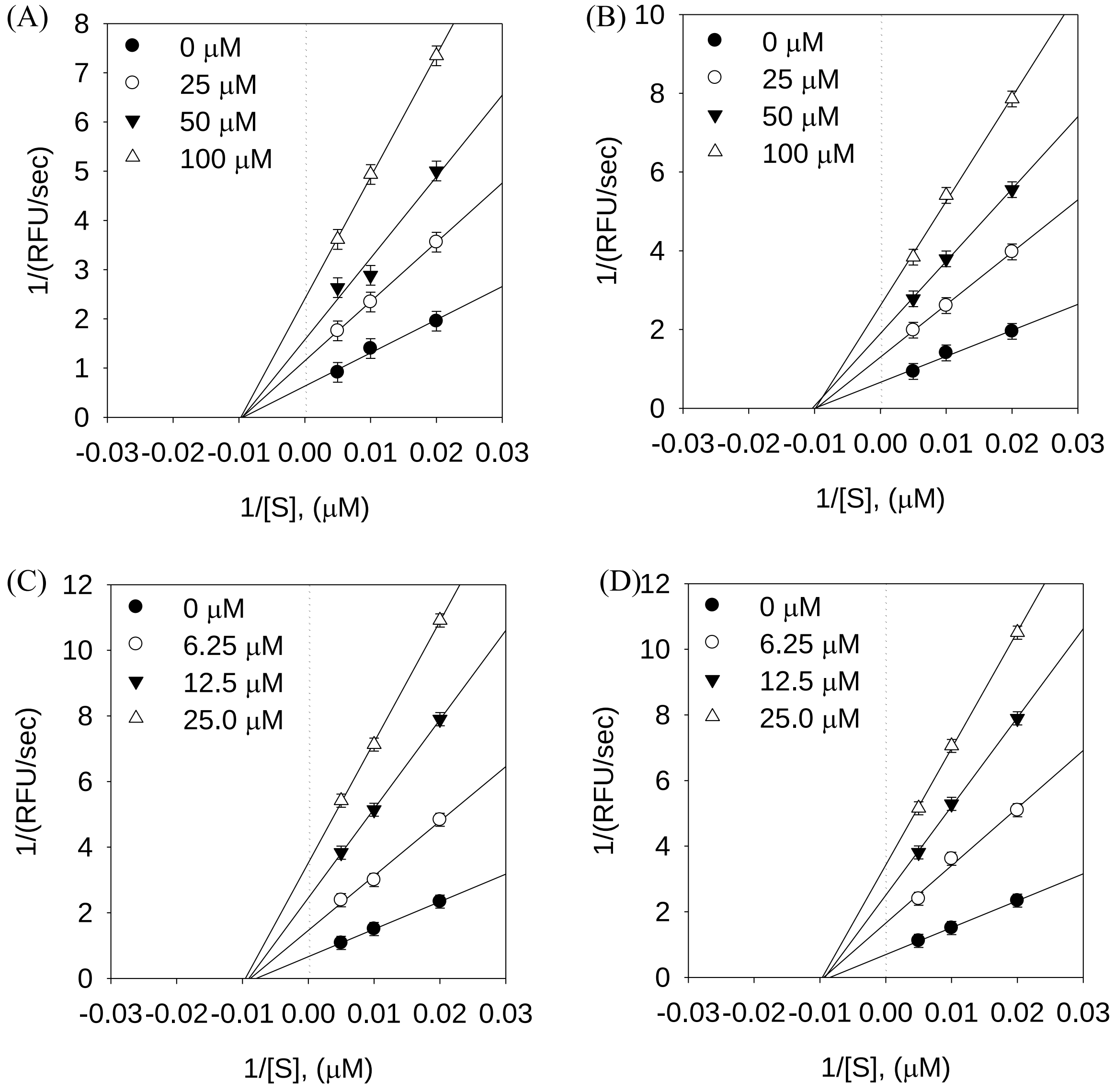

Figure S34. Lineweaver Burk plots of xanthones (A) **2**, (B) **3**, (C) **5**, and (D) **6** against BNA

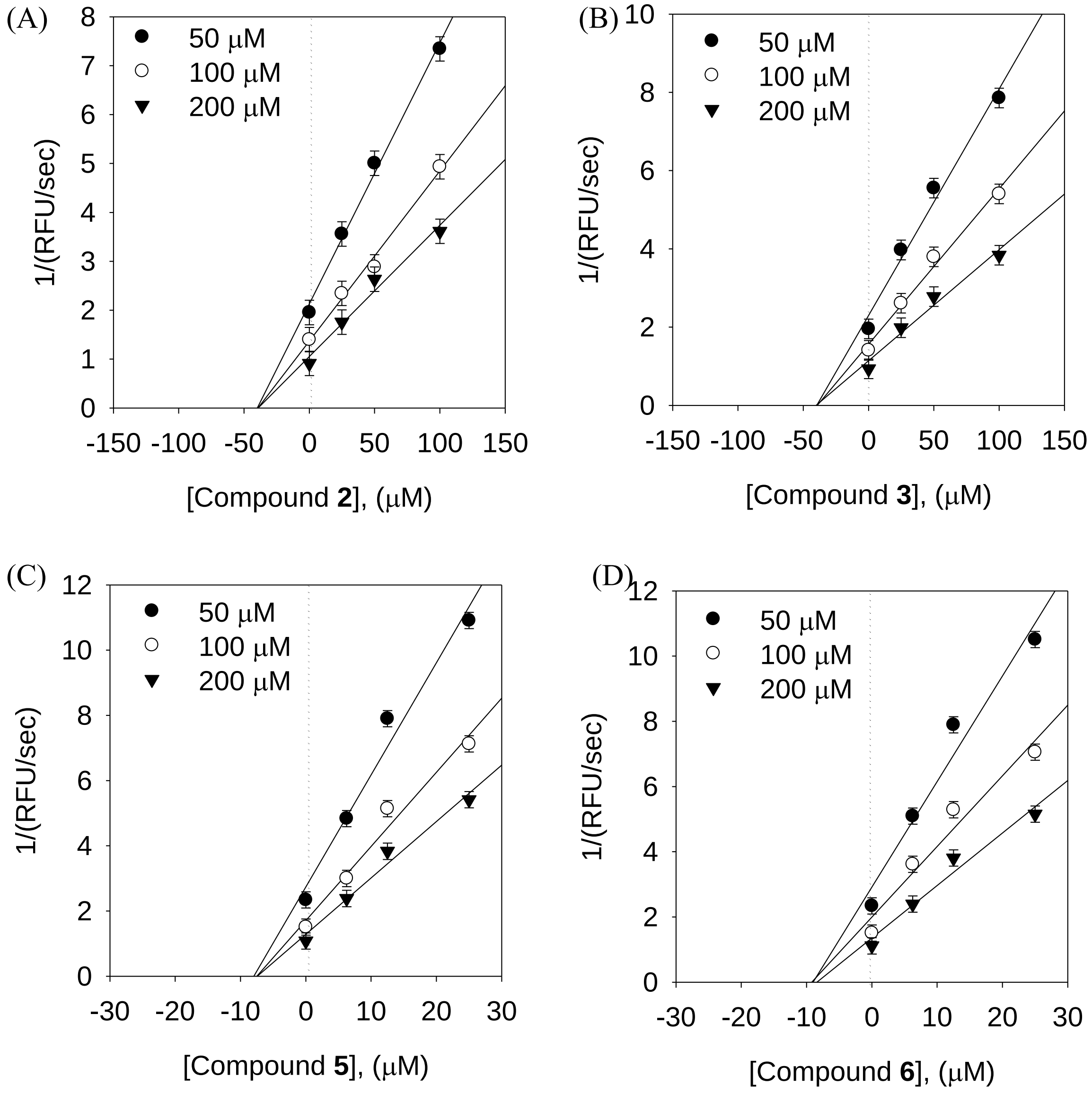

Figure S35. Dixon plots of xanthones (A) **2**, (B) **3**, (C) **5**, and (D) **6** against BNA

(A)

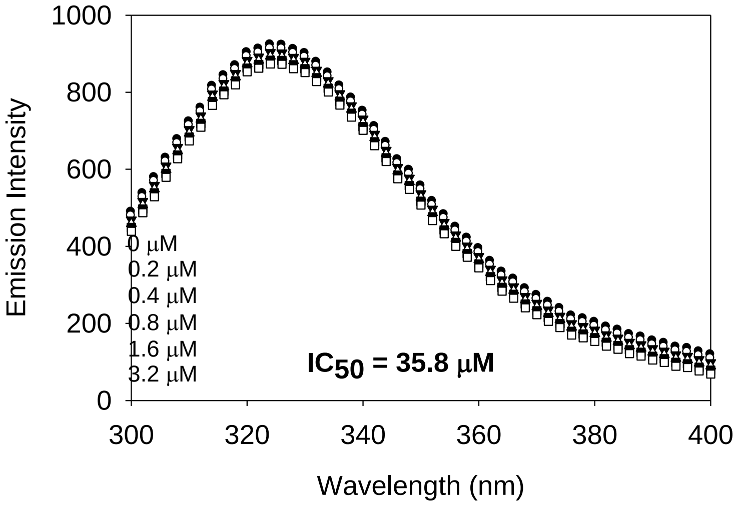

(B)

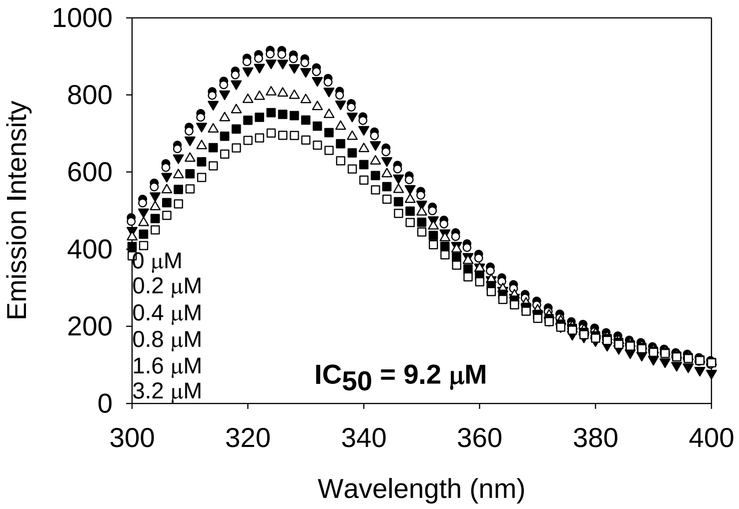

(C)

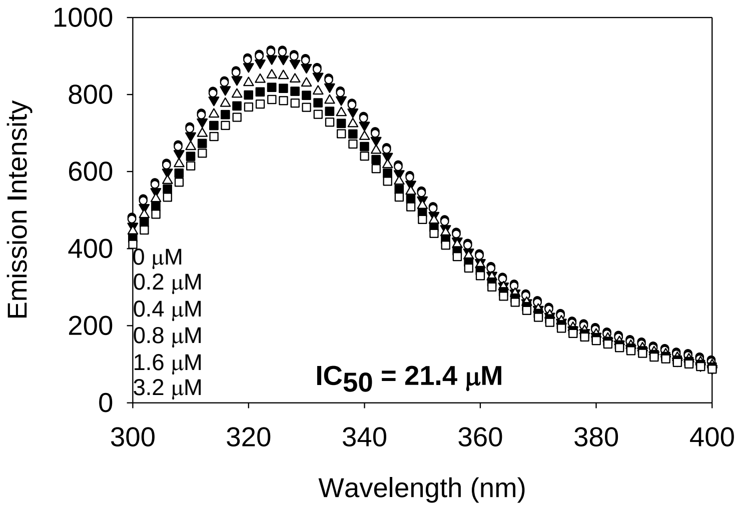

Figure S36. Fluorescence quenching effect of xanthones (A) **2**, (B) **6**, and (C) gentisein

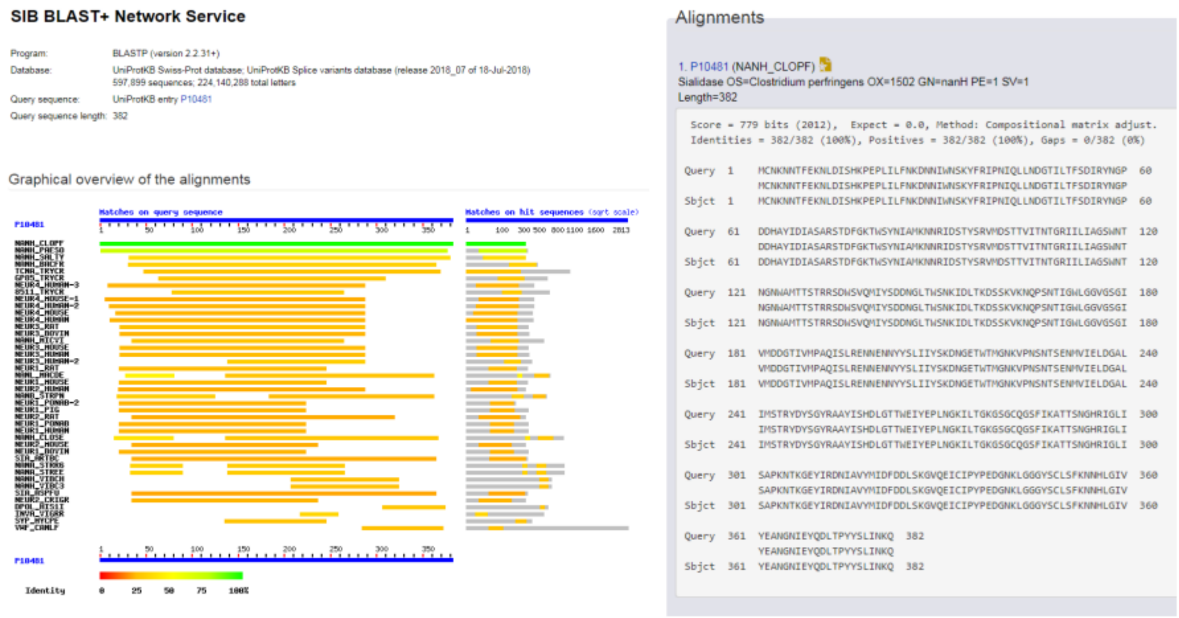

Figure S37. Fluorescence residues of neuraminidase from *Clostridium perfringens*. It was brought from ExPASy that is Swiss Institute of Bioinformatics. Trp31, 80, 118, 124, 135, 149, 172, 217, and 264. Tyr35, 57, 65, 82, 95, 141, 203, 204, 209, 246, 248, 251, 255, 267, 310, 318, 336, 347, 361, 369, 376, and 377. Phe8, 24, 36, 52, 76, 286, 322, and 352.

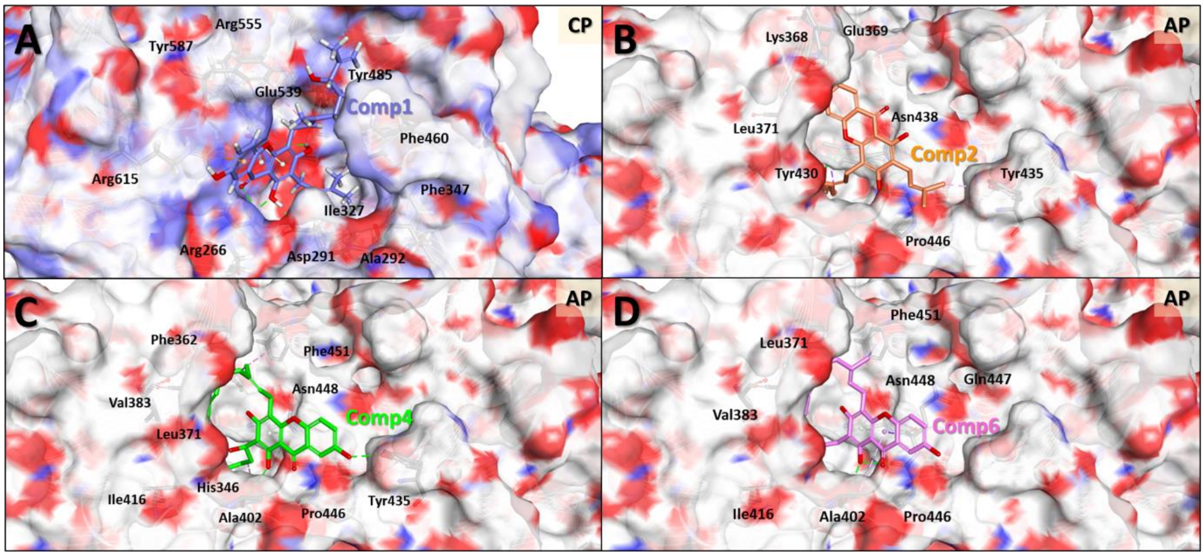

Figure S38. Predicted binding modes based on molecular docking (MD) structures between the four compounds and the *Clostridium perfringens* neuraminidase

Binding modes and molecular interactions of the compound **1** (A), **2** (B), **4** (C) and **6** (D) were displayed with the key residues in the binding site of the neuraminidase. Compound **1** (A) was docked in the catalytic pocket (CP), and the rest compounds of **2**, **4**, and **6** (B-D) were docked in the allosteric pocket (AP). Each panel used ball-and-stick model for the ligands to show the interaction details and the electrostatic surface model to show the binding pocket geometry of the protein.

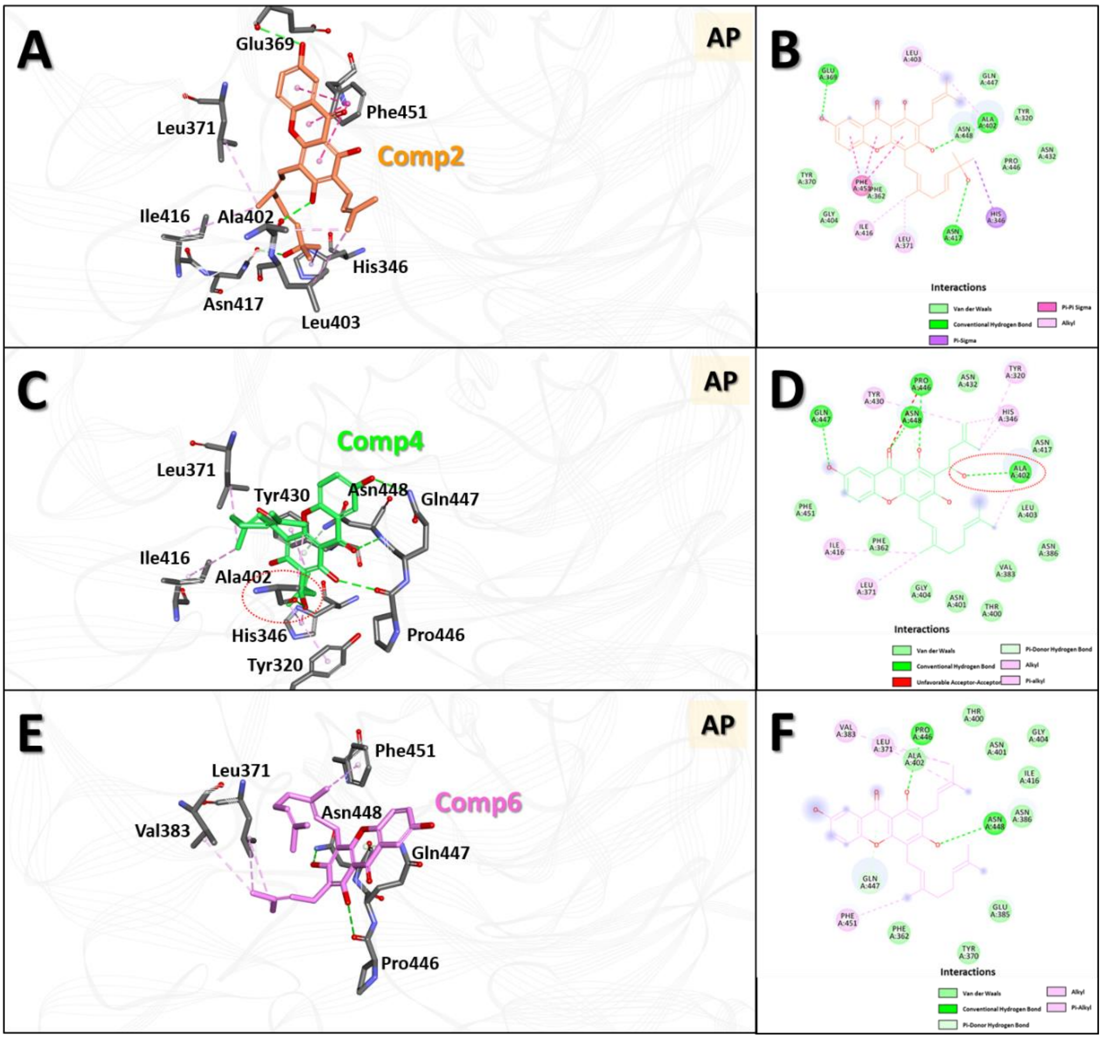

Figure S39. 3D and 2D binding modes of the three compounds in the allosteric pocket of the protein obtained by molecular dynamics simulation (MDS) studies

3D and 2D binding modes and molecular interactions of **1** (A, B), **2** (C, D) and **4** (E, F) were displayed with the key residues in the allosteric binding site of the neuraminidase. In panel A, C and E molecules are in ball-and-stick model to show clear intermolecular 3D interactions, while in panel B, D and F, in order to show entire interaction of the compound with the neighbor residues in the binding pocket of the protein, 2D molecular interaction diagrams were used. All the interactions are represented in dashed lines. The conventional hydrogen bonds are represented in green, carbon hydrogen bonds are shown in light green, π-sigma interactions are shown in purple, π-anion interactions are shown in orange, π-alkyl interactions are shown in pink respectively. The red dotted circle in panel D indicates the interaction part of the -OH group which is the only one difference between compounds **4** and **6**.
